# Generation of T-cell-redirecting bispecific antibodies with differentiated profiles of cytokine release and biodistribution by CD3 affinity tuning

**DOI:** 10.1038/s41598-021-93842-0

**Published:** 2021-07-13

**Authors:** Lauric Haber, Kara Olson, Marcus P. Kelly, Alison Crawford, David J. DiLillo, Richard Tavaré, Erica Ullman, Shu Mao, Lauren Canova, Olga Sineshchekova, Jennifer Finney, Arpita Pawashe, Supriya Patel, Ryan McKay, Sahar Rizvi, Ermelinda Damko, Danica Chiu, Kristin Vazzana, Priyanka Ram, Katja Mohrs, Amanda D’Orvilliers, Jenny Xiao, Sosina Makonnen, Carlos Hickey, Cody Arnold, Jason Giurleo, Ya Ping Chen, Courtney Thwaites, Drew Dudgeon, Kevin Bray, Ashique Rafique, Tammy Huang, Frank Delfino, Aynur Hermann, Jessica R. Kirshner, Marc W. Retter, Robert Babb, Douglas MacDonald, Gang Chen, William C. Olson, Gavin Thurston, Samuel Davis, John C. Lin, Eric Smith

**Affiliations:** 1 Regeneron Pharmaceuticals, Inc, Tarrytown, NY 10591 USA; 2 AstraZeneca, Cambridge, UK; 3 Lonza, Slough, UK

**Keywords:** Antibody therapy, Biotechnology, Cancer immunotherapy

## Abstract

T-cell-redirecting bispecific antibodies have emerged as a new class of therapeutic agents designed to simultaneously bind to T cells via CD3 and to tumor cells via tumor-cell-specific antigens (TSA), inducing T-cell-mediated killing of tumor cells. The promising preclinical and clinical efficacy of TSAxCD3 antibodies is often accompanied by toxicities such as cytokine release syndrome due to T-cell activation. How the efficacy and toxicity profile of the TSAxCD3 bispecific antibodies depends on the binding affinity to CD3 remains unclear. Here, we evaluate bispecific antibodies that were engineered to have a range of CD3 affinities, while retaining the same binding affinity for the selected tumor antigen. These agents were tested for their ability to kill tumor cells in vitro, and their biodistribution, serum half-life, and anti-tumor activity in vivo. Remarkably, by altering the binding affinity for CD3 alone, we can generate bispecific antibodies that maintain potent killing of TSA + tumor cells but display differential patterns of cytokine release, pharmacokinetics, and biodistribution. Therefore, tuning CD3 affinity is a promising method to improve the therapeutic index of T-cell-engaging bispecific antibodies.

## Introduction

Immunomodulatory approaches for cancer therapy have demonstrated promising clinical efficacy in recent years, leading to multiple approved therapeutics. Such therapeutics have focused on enhancing the T-cell response against tumors. Current approaches using traditional antibodies (bivalent and specific for the same antigen) that block T-cell checkpoint receptors such as CTLA-4 or PD-1 can result in durable responses in several tumor settings^1,2^.

Importantly, novel technologies such as engineered T cells^3^ and T-cell-activating bispecific antibodies (bsAbs) have also been approved^4–6^.

Tumor-specific antigens can be presented as peptides on the tumor cell surface in major histocompatibility complex (MHC) proteins, which interact with the T-cell receptors (TCR) on antigen-specific T cells to stimulate an anti-tumor response. BsAbs targeting CD3 are designed to bypass the TCR–MHC interaction normally required for TCR activation by co-engaging CD3 molecules on the T cell and a tumor antigen expressed on the surface of cancer cells. Currently, in addition to two approved CD3 bsAbs, several^7,8^ additional CD3-targeting bsAbs are in clinical development for the treatment of both hematologic and solid malignancies, with many others being investigated preclinically.

The efficacy, potency, and safety profiles of these bsAbs depend on multiple factors. For example, the valency and/or affinity of the tumor antigen targeting arm can influence its selectivity for tumor cells over normal tissue expressing the same target^9^. Analogously, the affinity for CD3 binding potentiates the level of T-cell activation that can affect both the potency as well as potential toxicity of a CD3 targeted bispecific. Herein, we generated and evaluated a series of CD3 bsAbs with altered binding affinity to CD3 that maintained unaltered binding to the tumor-cell-specific antigens (TSAs) across several distinct tumor targets (prostate specific membrane antigen [PSMA], Mucin-16 for ovarian cancer [MUC16], B-cell maturation antigen [BCMA], CD20). The resulting bsAbs exhibit differential cytotoxicity, cytokine release, biodistribution, and pharmacokinetic (PK) attributes that are hypothesized to translate clinically to an improved therapeutic window.

## Results

### Generation of a CD3-arm de-affinity maturation library yields variants with reduced affinity for CD3

**Figure 1:**
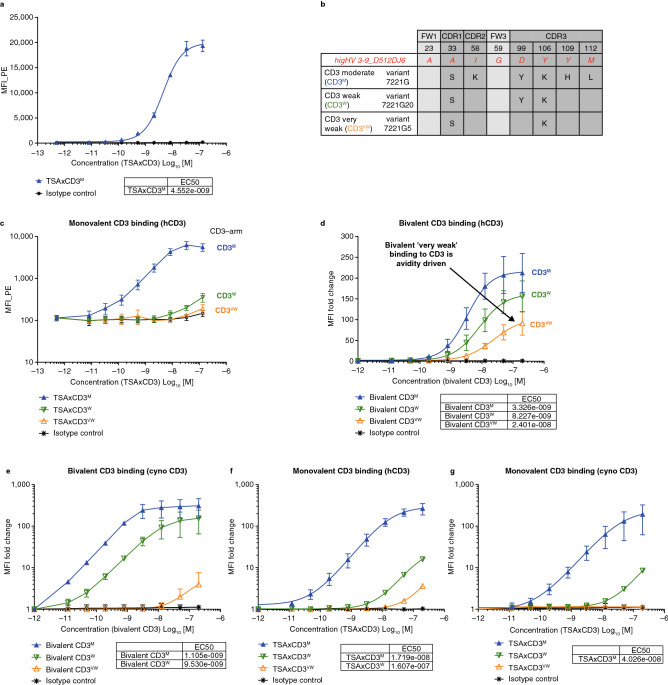
Generation of a CD3-arm de-affinity maturation library from VelocImmune mice-derived antibodies yields variants with reduced affinity for CD3. (**a**) Binding of anti-CD3 antibodies isolated from VelocImmune mice to Jurkat cells by flow cytometry; (**b**) summary of CD3 variant generated using germlining approach; (**c**) binding of CD3 variants to Jurkat cells by flow cytometry; (**d**) binding of selected parental CD3 variants to human T cells by flow cytometry; (**e**) binding of selected parental CD3 variants to cynomolgus T cells by flow cytometry; (**f**) binding of selected variants reformatted as TSAxCD3 bispecific antibodies on human T cells by flow cytometry; (**g**) binding of selected variants reformatted as TSAxCD3 bispecific antibodies on cynomolgus T cells by flow cytometry (three replicates). *CDR* complementary determining region; *EC50* half maximal effective concentration; *FW* framework region; *hCD3* human CD3; *m* moderate; *MFI* median fluorescent intensity; *TSA* tumor-cell-specific-antigen; *vw* very weak; *w* weak.

An antibody exhibiting a binding titration of moderate affinity to CD3 (7221G) was selected for further investigation and paired with a TSA-targeting arm (specifically the PSMA) to generate a bsAb as described previously (Supplementary Fig. S1)^10,11^. The resulting bsAb, as measured by flow cytometry, bound to Jurkat T cells via its CD3 arm with a half maximal effective concentration (EC50) value of approximately 5–10 nM (Fig. 1a). Anti-CD3 7221G variants (see methods for variant generation; Fig. 1b) were paired with the same TSA-targeting arm to generate bsAbs. Binding titration to Jurkat T cells was assessed using flow cytometry. All variants bound to Jurkat T cells with a range of estimated EC50 values higher than the bsAb paired with the ‘moderate’ parental 7221G CD3 arm (Fig. 1c; representative variants selected).

Besides 7221G (CD3^M^), two variant (7221G20 ‘weak’ [CD3^W^] and 7221G5 ‘very weak’ [CD3^VW^], these designations reflecting monovalent binding by flow cytometry) were chosen for further in-depth characterization. The parental, bivalent forms of these CD3 antibodies bound specifically to human T cells after incubation at 4 °C, with EC50 values ranging from 3 to 24 nM. A binding kinetics study by surface plasmon resonance at a physiologically relevant temperature (37 °C) demonstrated that the de-affinity maturation method altered both the binding association and dissociation rates of the chosen CD3^W^ and CD3^VW^ antibodies. CD3^M^ and CD3^W^ bound to cynomolgus T cells with EC50 values of 1 nM and 9.5 nM, respectively, and the binding from CD3^VW^ was too weak to allow for the calculation of EC50; the isotype-matched non-CD3 binding control antibody did not bind (Fig. 1d, e, Supplementary Figs. S2 and S3).

These CD3 arms were then paired with various TSA-targeting arms to generate bsAbs that target both solid and hematologic tumor-cell antigens (see Supplementary Fig. S1 for purification details, demonstrating uniformly high purity). PSMA and MUC16 were selected to represent targets of solid tumor malignancies, while CD20 and BCMA (B-cell maturation antigen) were selected for targets of hematologic malignancies (i.e. B-cell lymphoma and multiple myeloma, respectively)^12–16^. These bsAbs were monovalent for the CD3 binding arm, and the corresponding CD3^M^- and CD3^W^-based bsAbs specifically bound to human T cells with EC50 values ranging from 17 to 161 nM; the CD3^M^-based bsAb bound to cynomolgus T cells with an EC50 value of 40 nM, and the binding of the CD3^W^-based bsAb was detected but too weak to determine an EC50 value (representative data from bsAbs paired with a PSMA binding arm). The corresponding CD3^VW^-based bsAb bound very weakly to human T cells and its binding to cynomolgus T cells was not detectable by flow cytometry (Fig. 1f, g; Supplementary Fig. S2 and Table S1). Furthermore, the monovalent binding kinetics of the selected CD3^M^, CD3^W^, and CD3^VW^ arms to human CD3 were assessed at a physiologically relevant temperature (37 °C) by surface plasmon resonance (SPR; Supplementary Fig. S3). For the CD3^M^ and CD3^W^ arms the monovalent affinity values from these SPR studies align with the findings from the cell binding experiments. The SPR binding of the CD3^VW^ arm was too weak to allow for the calculation of binding kinetics which, taken together with the binding results from the bivalent form of the CD3^VW^ variant, suggest that the bivalent CD3^VW^ binding to CD3 is mainly driven by avidity.

The binding properties of the target arms (TSA arm) of these bsAbs were also validated by flow cytometry. As no changes were made to this arm, the monovalent binding EC50s to TSA-expressing cells were identical, completely independent of the CD3 binding arm (Supplementary Fig. S3). Finally, the exclusive binding specificity of the TSAxCD3 bsAbs to CD3 and their tumor antigen was assessed by flow cytometry, and no off-target binding was observed on the various negative cell lines tested (Supplementary Fig. S2).

### CD3 affinity drives the level of lysosomal/late endosomal accumulation of CD3 bsAbs

**Figure 2:**
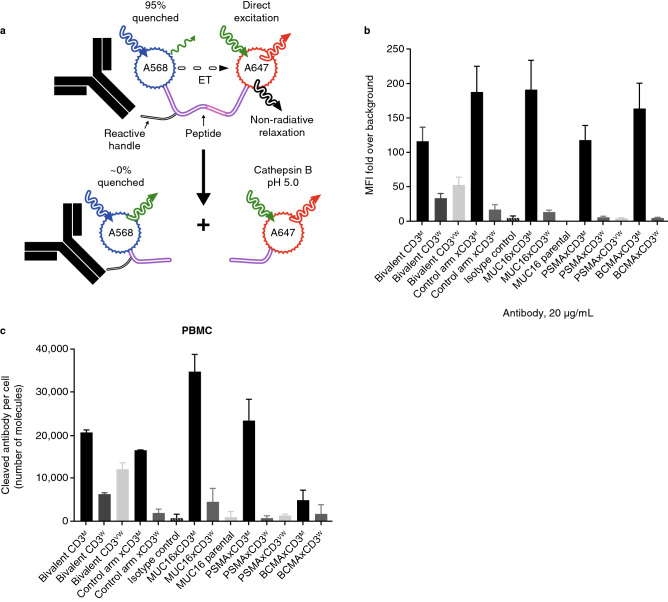
In vitro internalization by biosensor quantification. Antibodies were conjugated to a A647/568 biosensor. The biosensor molecule is composed of two fluorophores, A568 and A647, separated by a cleavable linker; when the molecule is intact, the A568 fluorophore is quenched by the A647 fluorophore (**a**), and binding (n = 2) of the conjugated antibody on the surface of a cell can be detected by flow cytometry using the A647 fluorophore (**b**). Upon internalization, the cleavable linker is cleaved by the lysosomal cysteine protease cathepsin B, and the remaining cleaved antibody can be detected by flow cytometry inside the cell for internalization measurement using the A568 fluorophore. PBMCs (**c**) were incubated with antibody biosensor conjugates for 18 h, stained with viability dye, and read in A568 channel by flow cytometry. The number of internalized and cleaved antibodies per cell in PBMCs was extrapolated from a A568-labeled beads standard curve (two replicates). (CD3^M^ arm shown in **black**, CD3^W^ arm shown in **dark gray**, CD3^VW^ arm shown in **gray**, Isotype control shown in **gray striped**). *BCMA* B-cell maturation antigen; *ET* energy transfer; *m* moderate; *MUC16* Mucin-16 for ovarian cancer; *PBMC* peripheral blood mononuclear cell; *PSMA* prostate specific membrane antigen; *vw* very weak; *w* weak.

We used a biosensor quantification method to assess the level of cathepsin B-dependent cleavage to mark the accumulation of parental CD3 antibodies or CD3-based bsAbs into the late endosomal/lysosomal compartments (Fig. 2a). The biosensor includes Alexa568 and Alexa647 fluorophores separated by a cathepsin B peptide substrate. When the biosensor is intact, Alexa568 is effectively quenched via efficient fluorescence resonance energy transfer (FRET) to Alexa647. When the cathepsin B peptide is cleaved by cathepsin B in the late endosomal/lysosomal compartments, the Alexa568 is detected, and the absolute numbers of cleaved molecules are determined. All antibodies tested were first conjugated to an A647/568 biosensor and binding to CD3 + T cells was assessed by flow cytometry (Fig. 2b).

When incubated with human peripheral blood mononuclear cells (PBMCs), we observed a higher level of biosensor cleavage from the parental CD3^M^ bivalent antibody and across all CD3^M^-based bsAbs when compared with weaker CD3 bivalent or bsAbs, regardless of their target antigen arm (Fig. 2c). These findings suggest that upon binding to CD3 on T cells, TSAxCD3 bsAbs were internalized and trafficked to cathepsin B-positive intracellular compartments, and that the degree of cleavage correlated with the affinity of the CD3 arm, i.e., the higher affinity CD3 trafficked more to the late endosomal/lysosomal compartments than the weaker affinity CD3, as the antigen-binding arm of the bsAbs was not engaged in this experiment.

### Target-dependent T-cell activation and cytotoxicity in vitro are CD3 affinity-dependent

The ability of TSAxCD3 bsAbs with varying CD3 binding affinity to activate TCR signaling was investigated in a NFAT-luciferase (NFAT-luc) cell-based bioassay using Jurkat T cells as reporter cells and various tumor cell lines. Solid tumor-targeting bsAbs, MUC16xCD3 and PSMAxCD3, were tested with OVCAR-3 and C4-2 cells, respectively. Whereas RAJI-CD80/CD86-negative and NCI-H929 cells were utilized with bsAbs targeting hematologic cancers, such as CD20xCD3 and BCMAxCD3, respectively.

**Figure 3:**
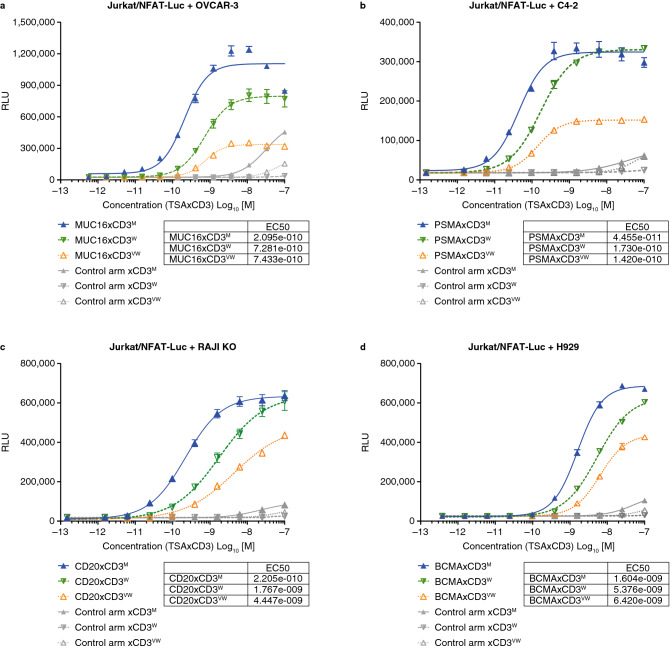
CD3-based bispecific antibodies induce in vitro activation of T cells in the presence of target cells, and activation is driven by the strength of the CD3 arm. (**a**) Jurkat/NFAT-luc cells were incubated with human MUC16-expressing OVCAR-3 cells and serial dilutions of MUC16xCD3 or CD3 bispecific control antibodies (gray symbols); (**b**) Jurkat/NFAT-luc cells were incubated with human PSMA-expressing C4-2 cells and serial dilutions of PSMAxCD3 or CD3 bispecific control antibodies (gray symbols); (**c**) Jurkat/NFAT-luc cells were incubated with human CD20-expressing RAJI-KO/CD80/CD86 cells and serial dilutions of CD20xCD3 or CD3-bispecific control antibodies (gray symbols); (**d**) Jurkat/NFAT-luc cells were incubated with human BCMA-expressing H929 cells and serial dilutions of BCMAxCD3 or CD3-bispecific control antibodies (gray symbols) (data in replicates of 2 or 3). *BCMA* B-cell maturation antigen; *EC50* half maximal effective concentration; *KO* knock-out; *luc* luciferase; *m* moderate; *MUC16* Mucin-16 for ovarian cancer; *PBMC* peripheral blood mononuclear cell; *PSMA* prostate specific membrane antigen; *RLU relative luciferase unit*; *TSA* tumor-cell-specific-antigen; *vw* very weak; *w* weak.

In all the systems tested, the TSAxCD3 bsAbs displayed an activation of the reporter cells in the presence of tumor cell lines expressing the desired human antigen. The stronger CD3 arms consistently showed increased potency values over the weaker CD3 arms (differences between the CD3^W^ and CD3^VW^ arms were context-dependent), suggesting a dependency on the affinity of binding of the CD3 arm (Fig. 3a–d). No activation was observed in control conditions where human CD3-mediated NFAT signaling was monitored in the absence of tumor antigen expression (Supplementary Fig. S4).

The cytotoxic potency of the TSAxCD3 bsAbs with differing CD3 affinities was assessed using a flow cytometry-based cytotoxicity assay^17^. BsAbs engineered to bind to MUC16, PSMA, or BCMA-expressing cells were evaluated for the ability to kill OVCAR-3, C4-2, or MOLP-8 cells, respectively. For each set of bsAbs, the tumor antigen targeting arms were identical for the studied target antigen but possessed a range of affinity to CD3 in their T-cell engaging arms.

**Figure 4:**
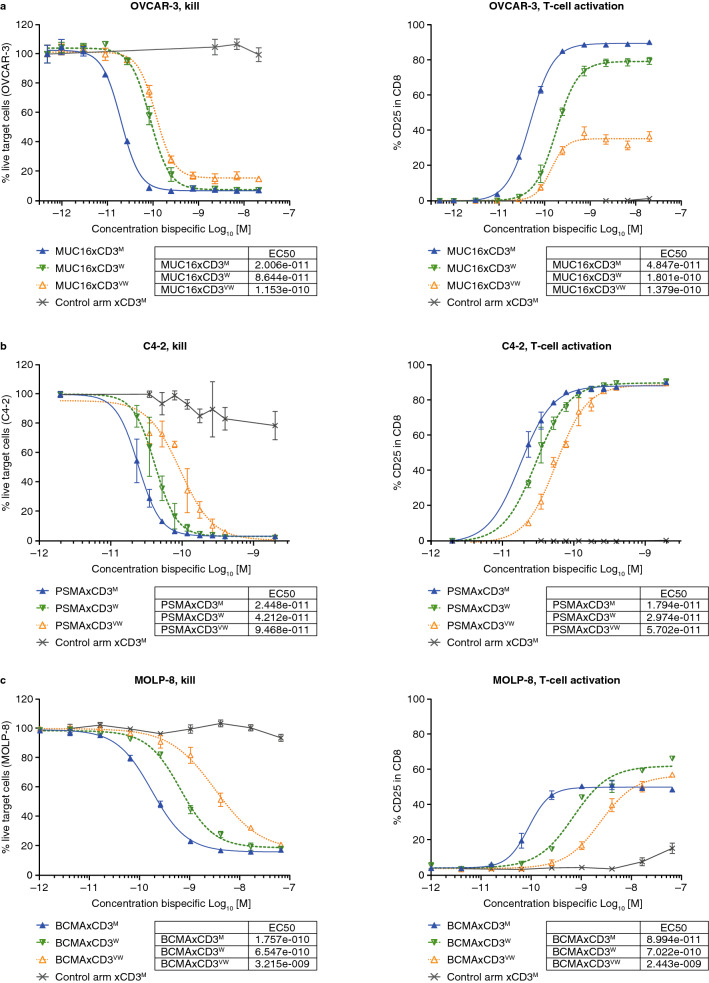
CD3-based bispecific antibodies are cytotoxic to tumor cells expressing the tumor antigen of interest, and the in vitro potency is driven by the strength of the CD3 arm. T-cell activation and cytotoxic potency of bispecific antibodies of various CD3 strengths were evaluated across various programs. The cytotoxic potency and T-cell activation induced by MUC16xCD3, PSMAxCD3, and BCMAxCD3 bispecific antibodies was evaluated against OVCAR-3 (**a**), C4-2 (**b**), and MOLP-8 (**c**) cells in the presence of human PBMCs, respectively (data representative of two independent experiments, three replicates). *BCMA* B-cell maturation antigen; *EC50* half maximal effective concentration; *m*, moderate; *MUC16* Mucin-16 for ovarian cancer; *PSMA* prostate specific membrane antigen; *vw* very weak; *w* weak.

The MUC16xCD3^M^ bsAb was the most potent at killing OVCAR-3 cells in the presence of human PBMCs. Although MUC16xCD3^M^ and MUC16xCD3^W^ were able to achieve complete killing of the target cells, the EC50 values for cell killing were 86 pM and 20 pM for the CD3^W^-based and CD3^M^-based bsAbs, respectively (Fig. 4a, left panel). The MUC16xCD3^VW^ bsAb exhibited the weakest potency (EC50 value of 115 nM) and was unable to achieve complete killing of the MUC16 + cells.

Next, PSMAxCD3^M^, PSMAxCD3^W^, and PSMAxCD3^VW^ bsAbs were tested in a cytotoxicity assay with C4-2 (PSMA +) cells in the presence of human PBMCs. Similar to the observations determined for the MUC16-based bsAbs, the PSMA + cells C4-2 were completely killed by all three PSMAxCD3 bsAbs, and the potency of cytotoxicity was again CD3 affinity-dependent, with calculated EC50 values ranging from approximately 20 pM to 90 pM (Fig. 4b, left panel).

Finally, to test CD3 binding affinity in vitro against hematologic tumor cells, BCMAxCD3^M^, BCMAxCD3^W^, and BCMAxCD3^VW^ bsAbs were evaluated in a cytotoxicity assay with MOLP-8 (BCMA +) cells in the presence of human PBMCs and results were similar to what was observed with the solid tumor-targeting bsAbs were observed. MOLP-8 cells (BCMA +) were completely killed by all three bsAbs, but the potency of cytotoxicity was also CD3 affinity-dependent, with EC50 values ranging from 180 pM to 3 nM (Fig. 4c, left panel).

The observed cytotoxic activity of human T cells against target-expressing cells also correlated with up-regulation of T-cell activation marker CD25, or PD-1 (Fig. 4a–c, right panels; Supplementary Fig. S5). Similar cytotoxic potency was observed in the presence of purified human T cells, with T-cell activation only observed in the presence of tumor cells expressing the targeted tumor antigen (Supplementary Fig. S5). These results demonstrate that the in vitro cytotoxic potency of T-cell-engaging bsAbs harboring identical tumor-antigen targeting arms is dependent on the affinity of their CD3 arm.

### The correlation between binding capacity on cancer cells and potency of CD3 bsAbs is CD3 affinity-dependent

**Figure 5:**
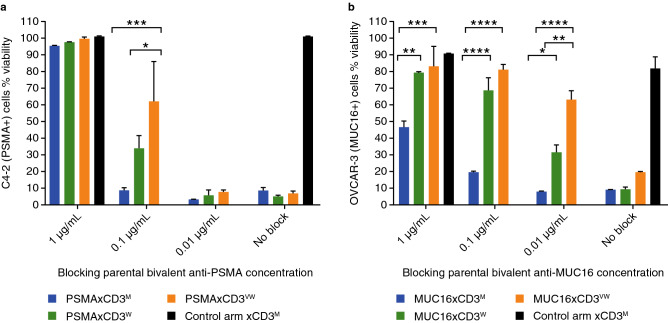
The correlation between target density on cancer cells and cytotoxic potency of CD3-based bispecific antibodies is CD3 affinity-dependent. The potency of PSMAxCD3 bispecific antibodies with moderate, weak, and very weak CD3 affinity was evaluated against C4-2 cells in the presence of human PBMCs and various concentrations of a blocking parental PSMA antibody (**a**). The potency of MUC16xCD3 bispecific antibodies with moderate, weak, and very weak CD3 affinity was evaluated against OVCAR-3 cells in the presence of human PBMCs and various concentrations of a blocking parental MUC16 antibody (**b**)**.** Two replicates, statistical analysis by ordinary two-way ANOVA with Tukey’s multiple comparisons test. *P < 0.02, **P < 0.007, ***P < 0.0008, and ****P < 0.0001. *ANOVA* analysis of variance; *m* moderate; *MUC16* Mucin-16 for ovarian cancer; *PBMC* peripheral blood mononuclear cell; *PSMA* prostate specific membrane antigen; *vw* very weak; *w* weak.

To evaluate the correlation between target antigen density and cytotoxic potency of CD3-based bsAbs based on their CD3 affinity, the three PSMAxCD3 or MUC16xCD3 bsAbs exhibiting a moderate, weak, or very weak CD3 affinity were utilized. These antibodies were tested in a cytotoxicity assay aimed at killing the PSMA + prostate cancer cell line C4-2 or the MUC16 + ovarian cancer cell line OVCAR-3 in the presence of human PBMCs. We hypothesized that the higher, avidity-driven binding affinity for the cancer antigens of the parental PSMA and MUC16 antibodies used to generate these bsAbs would block the target arm’s binding of the bsAbs in a dose-dependent manner. The titration of parental PSMA or MUC16 antibodies would then model a lowered target surface density of PSMA or MUC16 on the target cell line. In these experiments, three different concentrations of parental PSMA or MUC16 antibodies were tested (1 µg/mL, 0.1 µg/mL, and 0.01 µg/mL), as well as a control arm with no blocking antibodies. The concentration of TSAxCD3 bsAbs was kept constant (50 pg/mL) in these assays. The higher concentration of parental PSMA-blocking antibody (1 µg/mL) completely abolished the ability of CD3-based bsAbs to induce the killing of PSMA + C4-2 cells by human PBMCs, regardless of their CD3-arm binding affinity. In contrast, while pre-incubating the cells with 100 pg/mL or less of the parental antibody had no impact on the cytotoxic potency of the PSMAxCD3^M^ bsAb, the potency of the CD3^W^- and CD3^VW^-based bsAbs was already reduced in the presence of 100 pg/mL of parental PSMA antibody, with the CD3^VW^ arm being the most affected (Fig. 5a). In a similar manner, the higher concentration of parental MUC16-blocking antibody (1 µg/mL) reduced the maximum potency of the MUC16xCD3^M^ bsAb, but completely prevented MUC16xCD3^W^- and MUC16xCD3^VW^-mediated killing of OVCAR-3 cells. Pre-incubation with lower concentrations of the parental antibody had little or no impact on the potency of the MUC16xCD3^M^ bsAb, but significantly reduced the cytotoxic activity of the MUC16xCD3 bsAbs with weaker CD3 affinities (CD3^W^, CD3^VW^), again with CD3^VW^ being the most affected (Fig. 5b). These results demonstrate that, in conditions with reduced binding capacity to the TSA, a CD3-based bsAb with a stronger CD3 binding arm can better retain cytotoxic potency than those with a weaker CD3 binding arm.

### Biodistribution of bispecific T-cell-engaging antibodies is driven by the affinity of their CD3 arm

We investigated the in vivo distribution of Zirconium-89 (^89^Zr)-labeled MUC16xCD3^M^ and ^89^Zr-labeled MUC16xCD3^W^ bsAbs using positron emission tomography (PET) imaging in immunodeficient mice genetically humanized for both SIRPA and IL-15 (SRG-15 mice) and implanted with OVCAR-3 ascites cells and human PBMCs.

**Figure 6:**
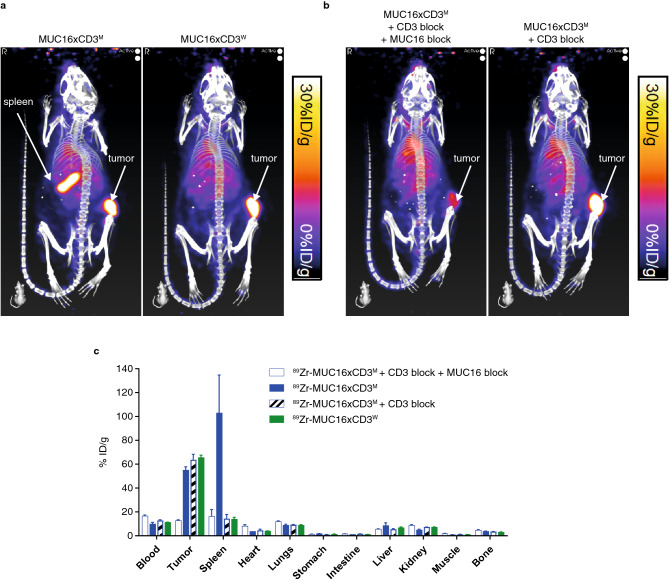
Biodistribution of bispecific T-cell-engaging antibodies is driven by the affinity of their CD3 arm. (**a**) Mice genetically humanized for SIRPA and IL-15 (SRG-15 mice) were implanted with 3 × 10^6^ OVCAR-3 ascites cells and 5 × 10^6^ human PBMCs and injected with ^89^Zr-radiolabeled antibodies at a final dose of 0.25 mg/kg via tail-vein injection when tumors were ~ 100 mm^3^; immune-PET images demonstrate the in vivo localization of the ^89^Zr radiolabeled antibodies 6 days post-dosing (**b**) in a control condition, mice were pre-treated with 10 mg/kg unlabeled bivalent MUC16 antibody and/or 10 mg/kg CD3-binding control antibody was administered in order to block binding of radiolabeled antibodies to MUC16 or CD3, respectively; (**c**) Quantitative ex vivo biodistribution analysis was performed following PET imaging on day 6 post-dosing. The tissue biodistribution values of the 89Zr-radiolabeled antibodies are presented as percent injected dose per gram (%ID/g). ^*89*^*Zr* Zirconium-89; *m*, moderate; *MUC16* Mucin-16 for ovarian cancer; *PBMC* peripheral blood mononuclear cell; *PET* positron emission tomography; *SIRPA* signal regulatory protein α; *vw* very weak; *w* weak.

The MUC16xCD3^M^ bsAb accumulated in both the spleen and MUC16-expressing tumor, suggesting recognition of CD3-positive T cells in the lymphoid tissue and MUC16 on the tumor cells. In contrast, the MUC16xCD3^W^ bsAb preferentially accumulated in the tumor site, but not in the spleen (Fig. 6a).

To further confirm that the tissue distribution of the bsAb is dependent on tumor-specific target and/or CD3 binding, the biodistribution of ^89^Zr-labeled MUC16xCD3^M^ was assessed in the presence of a saturating dose of unlabeled parental anti-CD3 and/or parental anti-MUC16 antibodies that block the binding of the radiolabeled bsAb. Pre-treatment with both parental antibodies resulted in the total lack of biodistribution of the ^89^Zr-labeled MUC16xCD3^M^ bsAb, whereas pre-treatment with parental CD3 antibody resulted in distribution to the tumor site, due to recognition of MUC16 on tumor cells (Fig. 6b). Levels of distribution were quantified ex vivo after the mice were euthanized (Fig. 6c), and these results confirmed the antibodies’ biodistribution as determined by immuno-PET imaging.

### CD3-based bsAbs with weaker CD3 arms efficiently reduced tumor burden while triggering a lower level of cytokines in xenogenic tumor models

To assess the in vivo antitumor activity of T-cell-redirecting bsAbs with different CD3 arm affinities, we established solid tumor and hematologic tumor xenograft models. First, we evaluated a tumor ascites model using OVCAR-3 ovarian cancer cells as previously described^17^. Treatment began on day 6 after tumor implantation with our panel of MUC16xCD3 bsAbs. In our second xenogenic model, MOLP-8 cells were co-implanted with human PBMCs and mice were treated prophylactically on day 0 with two different CD3-based bsAbs, each having the identical anti-BCMA arm and distinct anti-CD3 arms of either moderate or weak affinity. To evaluate the associated T-cell activation following bispecific addition, blood was collected 4 h post-treatment for analysis of cytokine release.

**Figure 7:**
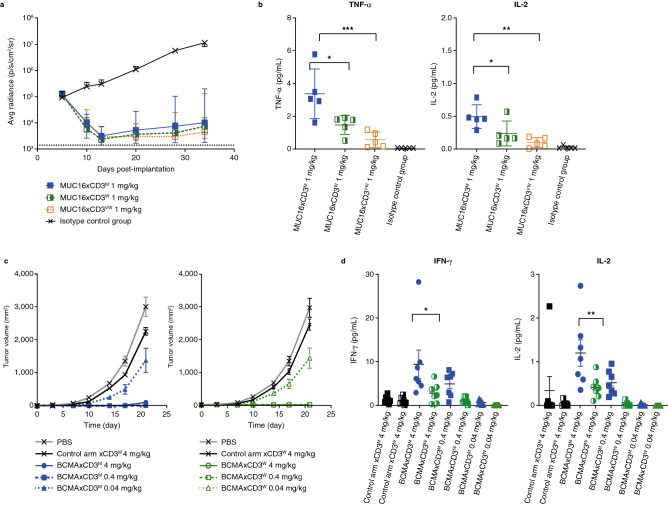
Reduction of tumor burden, unlike cytokine serum accumulation, is CD3 affinity-independent in xenogenic tumor models. (**a**) NSG mice were injected with 5 × 10^6^ human PBMCs before 1 × 10^6^ ascites cells from the OVCAR-3/luc cell line were administered IP (day 0). Mice were dosed IV with MUC16xCD3 bispecific antibodies or isotype control antibodies at 1 mg/kg, and imaged multiple times throughout the study to track tumor burden; (**b**) serum samples from the OVCAR-3/luc experiment were collected at 4 h post-dose to examine cytokine concentrations by MSD; (**c**) NSG mice were subcutaneously implanted with a mixture of human peripheral blood cells (1 × 10E^6^ cells) and MOLP-8 tumor cells (5 × 10E^6^ cells), and animals were continuously dosed IP twice weekly with BCMAxCD3 bispecific antibodies or controls through day 21, at various doses (4 mg/kg, 0.4 mg/kg, 0.04 mg/kg); (**d**) four hours post-implantation, serum from the MOLP-8 experiment was harvested for cytokine analysis by MSD. statistical analysis by ordinary one-way ANOVA with Tukey’s multiple comparisons test. *P < 0.05, **P < 0.002, and ***P < 0.0003. *Avg* average; *BCMA* B-cell maturation antigen; *d* day; *IP* intraperitoneally; *IV* intravenously*; luc* luciferase; *m* moderate; *MSD* Meso Scale Diagnostics; *MUC16* Mucin-16 for ovarian cancer; *NSG* NOD scid gamma; *PBMC* peripheral blood mononuclear cell; *PBS* phosphate-buffered saline; *vw* very weak; *w* weak.

In both xenogenic models, mice treated by all tumor-targeting CD3-based bsAbs had significantly reduced tumor burden compared with mice treated with control antibody. Importantly, the extent of tumor reduction was equally effective among TSAxCD3 bsAbs tested and was CD3 affinity-independent (Fig. 7a, c). All CD3-based bsAbs induced TNFα, IL-2, or IFNγ production compared with the isotype control antibody treatment. While cytokine production across the TSAxCD3-treated groups was low, it was notable that treatment with the strongest CD3-based bsAb generated the higher cytokine levels in both xenogenic studies (Fig. 7b, d). These data indicate that a reduction in CD3-arm binding affinity still allows for a potent antitumor response while limiting systemic cytokine levels.

### Bispecific T-cell-engaging antibodies with CD3 affinity-dependent pharmacokinetics achieved comparable potency in cynomolgus monkeys

To assess the impact of CD3 binding affinity on the PK characteristics of the panel of bsAbs with different CD3 binding affinities, PK studies in cynomolgus monkeys were performed with CD3-based bsAbs targeting CD20 (CD20xCD3^M^ and CD20xCD3^W^) or BCMA (BCMAxCD3^M^ and BCMAxCD3^W^). In both cases, TSAxCD3^M^ and TSAxCD3^W^ interacted with both the cynomolgus target antigen and cynomolgus CD3, and they bound with equal affinity to monkey TSA and differentially to CD3 (Supplementary Fig. S6).

**Figure 8:**
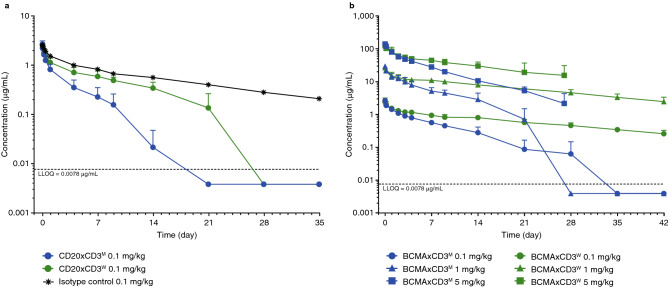
Pharmacokinetics. Total TSAxCD3^M^ and TSAxCD3^W^ in monkey serum. Concentrations of total CD20xCD3^M^ and CD20xCD3^W^ bispecific antibodies (**a**), or concentrations of total BCMAxCD3^M^ and BCMAxCD3^W^ bispecific antibodies (**b**) in cynomolgus monkey serum were measured using a non-validated ELISA. The LLOQ is 0.0078 μg/mL in neat monkey serum. *BCMA* B-cell maturation antigen; *ELISA* enzyme-linked immunosorbent assay; *LLOQ* lower limit of quantification; *m* moderate; *w* weak.

Following intravenous (IV) infusion of CD20xCD3^M^ and CD20xCD3^W^ to monkeys (n = 8/group), concentration–time profiles were characterized by an initial brief distribution phase, followed by a linear beta elimination phase and a terminal target-mediated elimination phase that was more predominant for CD20xCD3^M^ compared with CD20xCD3^W^. The steep drop in the terminal portions of the mean profiles may also be attributable in part to the presence of anti-drug antibodies impacting CD20xCD3^M^ and CD20xCD3^W^ concentrations, based on visual assessment of the individual PK profiles. The elimination half-lives (t_½_) of the antibodies, calculated from the majority of the elimination phase, were approximately 2.5 days and 6 days, respectively (Fig. 8a).

Dose-normalized maximum concentration (C_max_/dose) values were comparable among CD20xCD3^M^ and CD20xCD3^W^, as corresponding C_max_ values were within 1.0-fold between these dose groups. Dose-normalized exposure (Area under the concentration–time curve extrapolated to infinity (AUC_inf_)/dose) values were compared, demonstrating that CD20xCD3^W^ had a 2.6-fold greater exposure than that of CD20xCD3^M^. Consistent with the observed differential exposures, clearance (CL) values were 2.8-fold higher for CD20xCD3^M^ compared with CD20xCD3^W^ (Supplementary Table S2).

**Figure 9:**
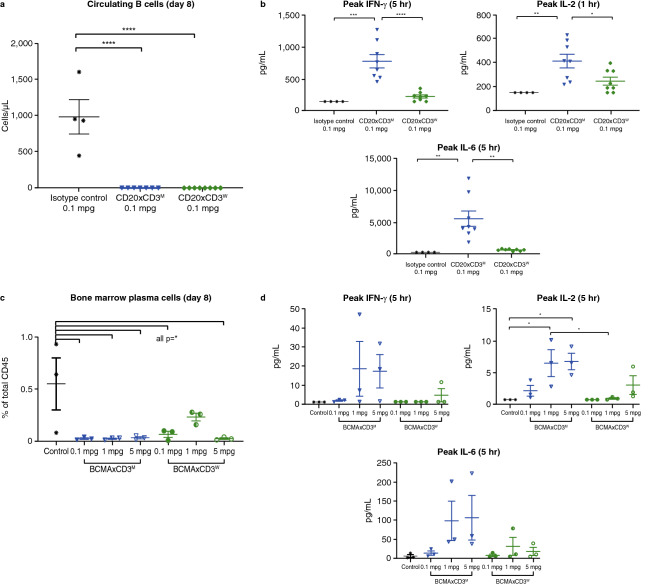
Potency of CD20xCD3 and BCMAxCD3 bispecific antibodies in cynomolgus monkeys. The number of circulating B cells after CD20xCD3 bispecific antibody treatment was determined by flow cytometry and reported as cells/µL blood (**a**), and peak cytokines were measured in serum by MSD and reported as pg/mL (**b**)**.** The number of bone marrow plasma cells after BCMAxCD3 bispecific antibody treatment was determined by flow cytometry and reported as percent of total CD45 + cells (**c**), and peak cytokines were measured in serum by MSD and reported as pg/mL (**d**). Statistical analysis by ordinary one-way ANOVA with Tukey’s multiple comparisons test. *P < 0.03, **P < 0.007, ***P < 0.0004, and ****P < 0.0001. *ANOVA* analysis of variance; *BCMA* B-cell maturation antigen; *MSD* Meso Scale Diagnostics.

To assess the potency of the CD20xCD3 bsAbs, their ability to deplete CD20 + B cells was determined by flow cytometry. Both CD20xCD3^M^ and CD20xCD3^W^ significantly depleted circulating and tissue CD20 + B cells 7 days after administration compared with isotype control (Fig. 9a; Supplementary Fig. S7). There was no major safety observation, and serum cytokines, including IFNγ, IL-2, and IL-6, were evaluated at multiple time points from 1 to 24 h post-IV infusion. Cytokines were elevated in CD20xCD3-dosed monkeys compared with isotype control, with CD20xCD3^M^ inducing higher peak cytokine levels compared with CD20xCD3^W^ (Fig. 9b; Supplementary Table S3).

In a second study, following IV bolus administration of BCMAxCD3^W^ to monkeys (n = 3/group), concentration–time profiles were characterized by an initial brief distribution phase followed by a single linear elimination phase throughout the 27-day (5 mg/kg group) and 42-day (0.1 and 1 mg/kg groups) study durations. For monkeys administered BCMAxCD3^M^ (n = 3/group), concentration–time profiles were characterized by an initial brief distribution phase, followed by a predominantly linear beta elimination phase and a subsequent non-linear terminal elimination phase at lower concentrations of BCMAxCD3^M^ in the 0.1 and 1 mg/kg dose groups. The steep drop in the terminal portions of the mean BCMAxCD3^M^ profiles may also be attributed in part to the presence of anti-drug antibodies. Calculation of half-life (t_1/2_) values over the majority of the elimination phase revealed a range of approximately 13–18 days for BCMAxCD3^W^, while BCMAxCD3^M^ ranged from approximately 4 to 5 days across the dose groups (Fig. 8b).

Consistent with the concentration–time profiles, dose-normalized exposure (C_max_/dose and AUC_inf_/dose) values were comparable (within 1.0- to 1.3-fold) for the respective 0.1, 1, and 5 mg/kg dose groups following BCMAxCD3^M^ and BCMAxCD3^W^ administration. However, the BCMAxCD3^W^ groups demonstrated approximately two- to threefold greater total exposure (AUC_inf_/dose) relative to the dose-matched BCMAxCD3^M^ groups (Supplementary Table S2b). Consistent with exposure, CL values were similar in the respective dose groups (ranging from ~ 8.4 to 9.1 mL/day/kg for BCMAxCD3^M^ and 2.9 to 4.4 mL/day/kg for BCMAxCD3^W^) and thus dose-independent across the respective IV dose groups.

To assess the potency of the BCMAxCD3 bsAbs, the ability of these bsAbs to deplete BCMA + plasma cells in the bone marrow was determined. Both BCMAxCD3^M^ and BCMAxCD3^W^ showed depletion of bone marrow CD38 + /CD138 + plasma cells 7 days after administration at all doses tested, without major safety observation (Fig. 9c; Supplementary Fig. S8). There was a trend toward cytokine elevation (IFNγ, IL-2, and IL-6) in the serum 5 h after dosing, with higher levels detected following the 1 mpk and 5 mg/kg dose compared with the 0.1 mg/kg dose for both bsAbs. BCMAxCD3^M^ induced higher amounts of cytokines than BCMAxCD3^W^ at all dose levels (Fig. 9d).

Collectively, in this second study, the relative dose-proportional increases in exposure indicated that BCMAxCD3^M^ and BCMAxCD3^W^ exhibited predominantly linear kinetics across all dose levels, with some evidence of non-linearity for BCMAxCD3^M^ at lower drug concentrations, likely due to the increased affinity of binding to CD3 that leads to enhanced target-mediated clearance. Higher relative exposures, longer half-lives and lower levels of cytokine release for the weak CD3-based compared with the moderate CD3-based bsAb suggest that the lower affinity to CD3 leads to more favorable kinetics; this may ultimately translate to comparable efficacy (TSA + cell depletion) with a lower production of cytokines and a more favorable dosing regimen (i.e. less frequent administration) in the clinic.

## Discussion

BsAbs are emerging as an important new class of therapeutic agents for cancer treatment. To date, two bsAbs have been approved for oncology indications (Removab for malignant ascites and Blincyto for lymphoblastic leukemia^4–6^), both of which are T-cell-redirecting bsAbs that engage CD3 and a tumor target antigen. Many additional T-cell-engaging bsAbs are currently in clinical and pre-clinical development across a range of indications; however, while these agents have shown considerable clinical promise, their observed efficacy is often accompanied by toxicities (e.g. CRS) that result from both the selection of the tumor-targeting antigen as well as from the T-cell response^18^. Thus, to achieve optimal efficacy for these agents, one needs to consider multiple factors, including not only the selection of molecular format and expression of tumor target, but also the binding affinity to those targets.

Several studies evaluating the outcome of modulating affinities of both the target and/or the effector arm of bsAbs have recently been published. Bortoletto et al.^19^ studied the effect of modulating the CD3 binding affinity of an EpCAMxCD3 bsAb and demonstrated that increasing the binding affinity to CD3 resulted in a lower capacity for the bsAb to target T cells against EpCAM-positive tumors. In contrast, their results suggest that decreasing the binding to CD3 was associated with efficient T-cell activation and cytotoxicity and, most importantly, even when used at low concentration. In this study, they generated differentiated CD3 binding affinities through the introduction of random mutations in the V_H_ CDR3 region of the CD3 binding arm, which raises the risk of introducing potential T-cell epitopes that could result in increased immunogenicity.

More recently, Mandikian et al.^20^ and Staflin et al.^21^ have extensively characterized the impact of CD3 and/or HER2 binding affinity on efficacy and nonclinical safety of a HER2xCD3 T-cell-dependent antibody. In contrast to the findings of Bortoletto et al., their data suggest that T-cell binding affinity had a limited impact on in vitro and in vivo antitumor activity. Besides finding a correlation between CD3 affinity and distribution to T-cell-rich tissues, they also observed that higher affinity for the tumor antigen (HER2) was associated with both optimized tumor-killing activity in mouse in vivo studies and a more severe toxicity profile in cynomolgus monkey studies, and suggested that a dose-fractionation strategy could increase the tolerability of such a bsAb.

In a separate study, the same group also reported CD3 affinity-related findings on a CD3 bsAb targeting CLL1, a hematologic cancer target^22^. In this study, they fixed the target arm to a high-affinity CD3 arm, resulting in an approximately 100-fold potency increase, but observed a less pronounced difference in potency in vivo that was attributed to more prolonged exposure of tumor to the lower CD3-affinity bispecific. While these studies demonstrated a correlation between CD3 affinity and in vitro potency, the in vivo observations were less clear. In addition, the bsAbs used in these studies were generated with distinct CD3 arms that were selected for their binding affinity, thus these CD3 antibody arms might engage different micro-epitopes on CD3, which may contribute to the observed differences in activity of the bsAbs.

In this study, we demonstrated that by reverting somatic hypermutations to their corresponding germline residues it is possible to generate bsAbs with a range of CD3 affinities that would be predicted to maintain the same CD3 binding epitope as well as presumed reduced risk of immunogenicity in contrast with methods that introduce novel amino acids not normally found in the CDR sequences. Because the germline reversions are the only changes introduced into the bsAbs used in this study, the changes in in vitro and in vivo properties demonstrated herein can be attributed to the alteration in CD3 binding affinity.

Using tumor cell lines derived from both solid and hematologic indications, we conducted in vitro cytotoxicity bioassays, observing that bsAbs with lower affinity for CD3 were less potent (higher EC50 values) than bsAbs with high affinity for CD3. This finding was consistent across different types of tumor cell lines when tested in vitro. In contrast, when used in the context of in vivo tumor models, CD3-based bsAbs with different CD3 affinities exhibited similar potencies. One potential explanation for this difference could be the duration of exposure of the tumor cells to T cells in the in vitro vs in vivo systems. A correlation has been observed in in vitro cytotoxicity assays whereby killing efficacy increases with the duration of the assay (Supplementary Fig. S8). This supports the notion that the efficacy differences observed in the in vitro systems between bsAbs with different CD3 affinities may be due to the shorter duration of those experiments compared with the in vivo tumor models.

The minimum copy number and density of antigen required for inducing an effective cell-killing response and the range where variations in antigen density can lead to a significant modification in cell-killing activity are likely unique for individual T-cell-engaging bsAbs^23^. The cytotoxic potency of PSMAxCD3 and MUC16xCD3 bsAbs was evaluated in an in vitro assay wherein a titration of blocking parental bivalent PSMA or MUC16 antibodies were used to decrease PSMA or MUC16 target availability. Under these conditions, we found that the bsAb engineered with the weakest anti-CD3 arm was the most affected. These findings suggest that potency differences due to modulation of CD3 affinity are more pronounced in the context of lower-density targets. Thus, a higher-affinity CD3-based bsAb may be preferred for the targeting of a tumor antigen expected to be expressed at very low density on the surface of cancer cells. Conversely, it could be theorized that a lower-affinity CD3 arm may be preferred in cases where tumor antigen expression is substantially higher than normal tissue expression, as that may lead to a better therapeutic index.

It is well established that CD3-based bispecific activity is associated with cytokine release syndrome (CRS), an important clinical toxicity^24,25^. We monitored the tumor burden and serum cytokines in mouse models of solid (MUC16-expressing) or hematologic (BCMA-expressing) malignancies following treatment with CD3-based bsAbs engineered with different CD3 affinities. In both in vivo models, and unlike our in vitro findings, efficacy was CD3-arm-affinity independent. However, the associated levels of T-cell-induced cytokine accumulation in serum correlated with the affinity of the CD3 arm. In both studies, the bsAb harboring the weaker CD3 arm induced lower levels of cytokines. Considering that in each study the bsAbs tested had identical tumor antigen binding-arm affinities, these data indicate that cytokine level reduction could be attained by tuning the CD3 affinity when designing a T-cell-redirecting bsAb.

There is existing evidence in the literature to support a correlation between CD3 affinity and distribution to T-cell-rich tissues, with higher CD3 affinity reducing systemic exposure and shifting bsAb distribution away from tumor to T-cell-containing tissues^21^. Using radiolabeled MUC16xCD3 bsAbs in mice pre-engrafted with human PBMCs and MUC16 + tumors, we studied the targeting of CD3-based bsAbs with various CD3-arm affinities. Minimal targeting to T-cell-rich organs, such as the spleen, was observed with a bispecific harboring a weak CD3 arm, which mainly targeted the MUC16 + tumor. In contrast, a bsAb harboring a CD3 arm with a higher affinity to CD3 targeted both T-cell-rich organs and the MUC16 + tumor. At the dose selected, we were able to achieve very similar levels of accumulation in the tumor with both bsAbs, suggesting that careful dosing strategy could result in the desired tumor targeting of a selected bsAb regardless of the affinity of its CD3 arm.

Pretreatment with an unlabeled anti-CD3 antibody blocked the targeting of the labeled antibody to the spleen, and pretreatment with both unlabeled CD3 and MUC16 antibodies blocked the targeting to both the spleen and the MUC16 + tumor, indicating that most of the targeting to the tumor is driven by the anti-MUC16 arm of the bsAb. The modular architecture of antibodies has been exploited to create many different bsAb formats. These formats vary in multiple ways, including their molecular weight, number of antigen-binding sites, spatial relationship between different binding sites, valency for each antigen, ability to support secondary immune functions, and PK half-life^26^.

For example, the structural difference between a bispecific T-cell engager (BiTE)^27^ and a bsAb that includes the half-life regulating Fc region leads to significant differences in the PK characteristics of these molecules, the former necessitating constant IV infusion whereas the latter are intended for extended intervals between infusions^28^. Non-human primate PK studies using bsAbs have been performed in order to predict clinical PK parameters in humans, with a direct impact on predicted clinical efficacy^29–31^. Direct binding experiments of monoclonal antibodies against CD3 or TCR resulted in progressive internalization and degradation of such antibodies^32^. Supported by our in vitro findings that bsAbs with greater affinity for CD3 were accumulated into late endosomal/lysosomal compartments more efficiently by T cells in the absence of target cells, we hypothesized that CD3 arm-affinity alteration in the context of a full-length, Fc-region-containing bsAb would also impact the PK of such molecules. In a non-human primate PK study, the dose-proportional increases in exposure indicate that CD3-based bsAbs with different affinities exhibited predominantly linear PK across all dose levels, with some evidence of non-linearity at lower concentrations for the bispecific harboring the stronger CD3 arm. The higher relative exposure, longer half-life, and comparable potency for the weak vs moderate CD3 affinity bsAb suggests that the lower-affinity CD3 binding leads to more favorable kinetics, which may translate to equal or better efficacy in the clinic but with a longer dosing interval and more favorable safety profile with respect to cytokine release.

Historically, in most cases of therapeutic antibody design, the strategy has been to obtain the highest achievable antigen-binding affinity^33,34^. However, our data suggest that in the case of a CD3-based bsAb, there are more parameters that need to be considered. These include the density of cell surface expression, kinetics of binding, and internalization rate of the two distinct binding targets.

We have demonstrated that modulation of the binding affinity of the effector arm, while keeping the tumor antigen binding arm affinity constant, results in the generation of bsAbs with distinct biodistribution, T-cell-induced cytokines, and PK properties. Each of these properties may influence the efficacy and safety profiles of such bsAbs, and ongoing human clinical trials comparing bsAbs with different CD3 affinities will help to fully characterize the therapeutic window of our bispecific platform and guide the design of future bsAbs on a case-by-case basis.

## Materials and methods

### Cell lines

The human cell lines used in this study were not directly derived from humans and were purchased from ATCC (American Type Culture Collection) or DSMZ (German Collection of Microorganisms and Cell Cultures):

- Jurkat: ATCC #TIB-152—T-cell lymphoblast established from peripheral blood
- OVCAR-3: ATCC #HTB-161—cell line established from the malignant ascites of a patient with adenocarcinoma of the ovary
- C4-2: ATCC #CRL-3314—human prostatic carcinoma cell line derived from human prostate cancer LNCap cells
- RAJI: ATCC #CCL-86—cell line established from lymphoblast-like cells
- H929: ATCC #CRL-9068—B lymphoblast cell line established from a malignant effusion in a patient with myeloma
- MOLP-8: DSMZ #ACC 569—multiple myeloma cell line established from a multiple myeloma patient.

### Generation of CD3 bsAb variants

In order to explore the consequence of altering CD3 affinity on CD3-based bsAb activity, we initially generated a panel of antibodies to CD3 using the VelocImmune mouse^35,36^. The moderate-affinity CD3 antibody 7221G was engineered to generate a library of binding affinity variants with the goal of reducing the CD3 binding affinity (Patent WO 2017/053856 A1). We targeted amino acid residues in the CDR regions and framework regions of the heavy chain that differed from germline sequences to generate the desired affinities while minimizing potential risk of immunogenicity. Antibody 7221G was determined to originate from the germline subgroup hIgHV 3-9_D512_J6, and we selected CDR residues (based on Kabat numbering) for reversion back to the corresponding germline amino acid residue based on that sequence. In total, 18 variants were generated using this ‘back to germline’ approach (Fig. 1b; Supplementary Table S4).

CD3 bsAbs were produced in CHO cells after transfection with three expression plasmids encoding a target antigen-binding heavy chain, a target antigen-binding light chain, and a CD3-binding heavy chain that contained the H_435_R, Y_436_F mutations as described previously^34,37^. BsAbs were purified by differential protein A-affinity chromatography. Plasmids encoding CD3 variants in the heavy chain variable domain were generated by mutagenesis using QuikChange II (Agilent Technologies) according to the manufacturer’s instructions.

### Binding to human and cynomolgus T cells by flow cytometry

Flow cytometric analysis was utilized to investigate binding of TSAxCD3 bsAbs to non-activated human or cynomolgus T cells. Briefly, human or cynomolgus monkey T cells were enriched from PBMCs using a human T-cell enrichment kit or a non-human primate pan T-cell isolation kit, respectively, following the manufacturer’s instructions (STEMCELL, Miltenyi Biotech, respectively). Post-enrichment, 2 × 10^5^ cells/well were incubated for 30 min at 4 °C with serial dilutions of TSAxCD3 bsAbs, parental CD3 antibodies, or an isotype-matched non-binding control. After incubation, the cells were washed twice with cold phosphate-buffered saline (PBS) containing 1% filtered fetal bovine serum (FBS), and an APC-conjugated anti-human secondary antibody, a PE-conjugated anti-CD4 antibody, and an AmCyan-conjugated anti-CD8 antibody were added to the cells. The plates were incubated for an additional 30 min. After incubation, cells were washed, re-suspended in 200 μL cold PBS containing 1% filtered FBS and analyzed by flow cytometry on a BD FACS Canto II.

### Binding to cynomolgus B cells by flow cytometry

PBMCs were isolated from cynomolgus monkey whole blood, lysed with PharmaLyse (BD), blocked with 20% mouse serum, and stained with CD20xCD3 or isotype control for 45 min on ice. Cells were washed and stained with in-house AlexaFluor647 conjugated anti-human secondary antibody (2.8 ug/test) as well as a phenotyping antibody cocktail including HLA-DR FITC, CD16 PE-Cy7, CD40 PE, CD8 BV786, CD14 BV421, CD2 BV510, CD45 BUV396, and CD4 BUV737. After washing, cells were analyzed on the BD Fortessa flow cytometer. Binding to B cells was determined by evaluating the mean fluorescence of Alexa647 on CD45 + /CD14-/CD2-/CD16-/HLA-DR + /CD40 + cells.

Measured values were analyzed using a four-parameter logistic equation over a multi-point response curve using GraphPad Prism version 7.0e for Mac OS X, GraphPad Software, San Diego, California USA, www.graphpad.com.

### In vitro internalization by biosensor quantification

The in vitro internalization of CD3 antibodies was measured using a Biosensor quantification method. Briefly, antibodies were conjugated to a A647/568 biosensor, and human PBMCs were incubated with 10 µg/mL of antibody biosensor conjugates for 18 h. The cells were then stained with a violet viability dye (Thermo L34963) and analyzed by flow cytometry. Anti-human IgG beads (Bangs Lab 816A) were stained with 10 µg/mL of a A568-labeled antibody for 30 min and analyzed by flow cytometry in order to generate a standard curve. PBMCs were gated for viable single events to obtain median fluorescent intensity (MFI) in the A568 channel. All MFIs were first background-corrected against unlabeled cells or beads, and dye antibody ratio-corrected. The number of internalized and cleaved antibodies per cell in PBMCs or purified T cells was extrapolated from the A568-labeled beads standard curve.

Measured values were reported using GraphPad Prism version 7.0e for Mac OS X, GraphPad Software, San Diego, California USA, www.graphpad.com.

### Nuclear factor of activated T cells-luciferase (NFAT-luc) cell-based reporter bioassay

See method in supplementary methods.

### In vitro flow cytometry cytotoxicity assays using human effector cells

The specific killing of human and monkey TSA-expressing target cells by naïve human effector cells was assessed by flow cytometry.

For studies using solid tumor cells lines, OVCAR-3, C4-2 cells were labeled with 1 μM of Violet Cell Tracker (Life Sciences) fluorescent tracking dye. After labeling, cells (5000–10,000 cells/well) were plated overnight at 37 °C in complete media (RPMI supplemented with 10% FBS, 100 U/mL penicillin, 100 µg/mL streptomycin, 292 µg/mL L-glutamine). Separately, human PBMCs (or T cells from the same donor, purified using STEMCELL kit cat#17951 and following the manufacturer’s protocol) were plated in complete media at 1 × 10^6^ cells/mL and incubated overnight at 37 °C. During this step, human PBMCs were enriched for lymphocytes by depleting adherent macrophages, dendritic cells, and some monocytes. The next day, target cells were co-incubated with adherent cell-depleted naïve PBMCs or purified T cells (effector: target cell ratio of 4:1 for human PBMCs, 2:1 for purified T cells) and serial dilution of relevant TSAxCD3 bsAbs or a CD3-binding control for 48–72 h at 37 °C in complete media. Cells were removed from cell culture plates using an enzyme-free cell dissociation buffer and analyzed by flow cytometry. For flow cytometry analysis, cells were stained with a live/dead far-red cell tracker. Counting beads were added to each well immediately before flow cytometry analysis, and 1 × 10^5^ beads were collected for each sample. For assessment of killing specificity, cells were gated on live Violet-labeled populations. Percent live population was recorded and used for the calculation of survival.

T-cell activation was assessed by incubating cells with directly conjugated antibodies to CD2, CD4, CD8, and CD25, and by reporting the percent of activated (CD25^+^) T cells out of CD8 + T cells.

Measured values were analyzed using a four-parameter logistic equation over a multi-point response curve using GraphPad Prism version 7.0e for Mac OS X, GraphPad Software, San Diego, California USA, www.graphpad.com.

For the hematologic tumor cell lines study, see method in supplementary methods.

### Parental blocking cytotoxicity assay

See method in supplementary methods.

### Xenogenic tumor model

All animal studies were approved by Regeneron’s Institutional Animal Care and Use Committee (IACUC) and performed in accordance with its relevant guidelines and regulations; additionally, studies were carried out in accordance with the ARRIVE guidelines.

#### OVCAR-3 bioluminescence imaging (BLI) study

Eight-week-old NOD SCID gamma chain knock-out (NSG) mice (Jackson Laboratory) were injected with 5 × 10^6^ human PBMCs (ReachBio) before 1 × 10^6^ ascites cells from the OVCAR-3/luc cell line, previously passaged in vivo*,* were administered intraperitoneally (IP; day 0). Mice were checked for T-cell engraftment by flow cytometry then assigned to groups using BLI to ensure similar tumor burden. Mice were dosed IV at days 6, 10, and 13 with MUC16xCD3 bsAbs or isotype control antibodies at 1 mg/kg. Mice were imaged multiple times throughout the study to track tumor burden.

Serum samples were collected at 4 h post-dose to examine cytokine concentrations by Meso Scale Diagnostics (MSD; Rockville, MD).

#### MOLP-8 prophylactic admix model

NSG mice were subcutaneously implanted with a mixture of human peripheral blood cells (1 × 10^6^ cells) and MOLP-8 tumor cells (5 × 10^6^ cells) after verifying BCMA expression on cultured tumor cells. Four hours post-implantation, serum was harvested for cytokine analysis by MSD. Animals were continuously dosed IP twice weekly through day 21, at various doses (4 mg/kg, 0.4 mg/kg, 0.04 mg/kg).

#### Statistical analyses

Statistical analyses were performed using GraphPad Prism version 7.0e for Mac OS X, GraphPad Software, San Diego, California USA, www.graphpad.com. Statistical significance for cytokine analysis was determined by ordinary one-way ANOVA with Tukey’s multiple comparisons post-test.

### Immuno-PET imaging

MUC16xCD3^M^ and MUC16xCD3^W^ antibodies were deglycosylated with PNGase F and subsequently conjugated to desferrioxamine (DFO) at glutamine 295 via microbial transglutaminase-mediated transamidation^38^. PET radionuclide ^89^Zr was then chelated into the DFO-conjugated antibodies as previously described^39^. Velocigene mice humanized for SIRPA and IL-15 (SRG-15) were implanted subcutaneously with 3 × 10^6^ OVCAR-3 ascites cells alongside the IP implantation of 5 × 10^6^ human PBMCs^40^. When OVCAR-3 tumors were ~ 100 mm^3^, mice received ^89^Zr-radiolabeled antibodies at a final dose of 0.25 mg/kg via tail-vein injection. In some groups, 10 mg/kg unlabeled bivalent MUC16 antibody or 10 mg/kg CD3-binding control antibody was administered in order to block binding of radiolabeled antibodies to MUC16 or CD3, respectively.

Immuno-PET imaging was performed over 6 days post-dosing to assess the in vivo localization of the ^89^Zr-radiolabeled antibodies. PET and computed tomography (CT) imaging were performed using a pre-calibrated Sofie Biosciences G8 PET/CT instrument (Sofie Biosciences and Perkin Elmer). The energy window ranged from 150 to 650 keV with a reconstructed resolution of 1.4 mm at the center of the field of view. Mice underwent induction anesthesia using isoflurane before imaging and were kept under continuous flow of isoflurane during 10-min static PET acquisitions. CT images were acquired directly following PET acquisition. The PET image was subsequently reconstructed using preconfigured settings. Decay-corrected PET data and CT data were processed using VivoQuant software (inviCRO Imaging Services, Boston MA, version 4.0, www.vivoquant.com) into false-colored co-registered PET-CT maximum-intensity projections on a color scale calibrated to indicate a signal range of 0 to 30% of injected dose per volume, expressed as %ID/g.

For ex vivo biodistribution analysis, mice were euthanized following imaging on day 6 post-dosing. Blood was collected via cardiac puncture and placed into pre-weighed counting tubes. Normal tissues (spleen, heart, lungs, stomach, small intestine, liver, kidneys, and bone) and OVCAR-3 tumors were excised and placed into individual pre-weighed counting tubes. All tubes were subsequently re-weighed to determine the weight of the blood and tissues. The γ-emission radioactivity for all samples at 511 keV were then measured on an automatic gamma counter (Hidex AMG) and results reported in counts per minute. The %ID for each sample was determined using sample counts relative to dose-standard counts prepared from the original injected antibodies. Subsequently, the individual %ID/g values were derived by dividing the %ID value by the respective weight of the appropriate blood, tissues, or tumor samples.

Ex vivo biodistribution values were reported using GraphPad Prism version 7.0e for Mac OS X, GraphPad Software, San Diego, California USA, www.graphpad.com.

### Cynomolgus pharmacokinetic studies

See method in supplementary methods.

### Binding kinetics by surface plasmon resonance

See method in supplementary methods.

## Supplementary Information

Supplementary Information 1.

## References

[1] Park YJ, Kuen DS, Chung Y (2018). Future prospects of immune checkpoint blockade in cancer: From response prediction to overcoming resistance. Exp. Mol. Med..

[2] Marin-Acevedo JA (2018). Next generation of immune checkpoint therapy in cancer: New developments and challenges. J. Hematol. Oncol..

[3] June CH, O’Connor RS, Kawalekar OU, Ghassemi S, Milone MC (2018). CAR-T cell immunotherapy for human cancer. Science.

[4] Przepiorka D (2015). FDA approval: Blinatumomab. Clin. Cancer Res..

[5] Ruf P (2010). Pharmacokinetics, immunogenicity and bioactivity of the therapeutic antibody catumaxomab intraperitoneally administered to cancer patients. Br. J. Clin. Pharmacol..

[6] Seimetz D, Lindhofer H, Bokemeyer C (2010). Development and approval of the trifunctional antibody catumaxomab (anti-EpCAM x anti-CD3) as a targeted cancer immunotherapy. Cancer Treat. Rev..

[7] Velasquez MP, Bonifant CL, Gottschalk S (2018). Redirecting T cells to hematological malignancies with bispecific antibodies. Blood.

[8] Yu S (2017). Recent advances of bispecific antibodies in solid tumors. J. Hematol. Oncol..

[9] Mazor Y (2017). Enhanced tumor-targeting selectivity by modulating bispecific antibody binding affinity and format valence. Sci. Rep..

[10] Smith E (2015). A novel, native-format bispecific antibody triggering T-cell killing of B-cells is robustly active in mouse tumor models and cynomolgus monkeys. Sci. Rep..

[11] Dilillo DJ (2021). A BCMAxCD3 bispecific T cell-engaging antibody demonstrates robust antitumor efficacy similar to that of anti-BCMA CAR T cells. Blood Adv..

[12] Chang SS (2004). Overview of prostate-specific membrane antigen. Rev. Urol..

[13] Hiss D (2012). Optimizing molecular-targeted therapies in ovarian cancer: The renewed surge of interest in ovarian cancer biomarkers and cell signaling pathways. J. Oncol..

[14] Kwatra MM (2017). A rational approach to target the epidermal growth factor receptor in Glioblastoma. Curr. Cancer Drug Targets.

[15] Meerten TV, Hagenbeek A (2009). CD20-targeted therapy: A breakthrough in the treatment of non-Hodgkin’s lymphoma. Neth. J. Med..

[16] Tai Y, Anderson KC (2015). Targeting B-cell maturation antigen in multiple myeloma. Immunotherapy.

[17] Crawford A (2019). A mucin 16 bispecific T cell-engaging antibody for the treatment of ovarian cancer. Sci. Transl. Med..

[18] Dahlén E, Veitonmäki N, Norlén P (2018). Bispecific antibodies in cancer immunotherapy. Ther. Adv. Vaccin. Immunother..

[19] Bortoletto N, Scotet E, Myamoto Y, D’Oro U, Lanzavecchia A (2002). Optimizing anti-CD3 affinity for effective T cell targeting against tumor cells. Eur. J. Immunol..

[20] Mandikian D (2018). Relative target affinities of T-cell-dependent bispecific antibodies determine biodistribution in a solid tumor mouse model. Mol. Cancer Ther..

[21] Staflin K (2020). Target arm affinities determine preclinical efficacy and safety of anti-HER2/CD3 bispecific antibody. JCI Insight.

[22] Leong SR (2017). An anti-CD3/CLL-1 bispecific antibody for the treatment of acute myeloid leukemia. Blood.

[23] Ellerman D (2019). Bispecific T-cell engagers: Towards understanding variables influencing the in vitro potency and tumor selectivity and their modulation to enhance their efficacy and safety. Methods.

[24] Teachey DT (2013). Cytokine release syndrome after blinatumomab treatment related to abnormal macrophage activation and ameliorated with cytokine-directed therapy. Blood.

[25] Maude SL, Barrett D, Teachy DT, Grupp SA (2014). Managing cytokine release syndrome associated with novel T cell-engaging therapies. Cancer J..

[26] Spiess C, Zhai Q, Carter PJ (2015). Alternative molecular formats and therapeutic applications for bispecific antibodies. Mol. Immunol..

[27] Wang Q (2019). Design and production of bispecific antibodies. Antibodies.

[28] Ferl GF (2018). A preclinical population pharmacokinetic model for anti-CD20/CD3 T-cell dependent bispecific antibodies. Clin. Transl. Sci..

[29] Betts A (2019). A translational quantitative systems pharmacology model for CD3 bispecific molecules: application to quantify T cell-mediated tumor cell killing by P-Cadherin LP DART. AAPS J..

[30] Chen X (2016). Mechanistic projection of first in human dose for bispecific immunomodulatory P-Cadherin LP-DART: An integrated PK/PD modeling approach. Clin. Pharmacol. Ther..

[31] Saber H, Del Valle P, Ricks TK, Leighton JK (2017). An FDA oncology analysis of CD3 bispecific constructs and first-in-human dose selection. Regul. Toxicol. Pharmacol..

[32] Schaffar L, Dallanegra A, Breittmayer JP, Carrel S, Fehlmann M (1988). Monoclonal antibody internalization and degradation during modulation of the CD3/T-cell receptor complex. Cell. Immunol..

[33] Carter PJ (2006). Potent antibody therapeutics by design. Nat. Rev. Immunol..

[34] Lippow SM, Wittrup KD, Tidor B (2007). Computational design of antibody-affinity improvement beyond in-vivo maturation. Nat. Biotechnol..

[35] Murphy AJ (2014). Mice with megabase humanization of their immunoglobulin genes generate antibodies as efficiently as normal mice. Proc. Natl. Acad. Sci. U. S. A..

[36] Macdonald LE (2014). Precise and in situ genetic humanization of 6 Mb of mouse immunoglobulin genes. Proc. Natl. Acad. Sci. U. S. A..

[37] Tustian AD, Endicott C, Adams B, Mattila J, Bak H (2016). Development of purification processes for fully human bispecific antibodies based upon modification of protein A binding avidity. MAbs.

[38] Jeger S (2010). Site-specific and stoichiometric modification of antibodies by bacterial transglutaminase. Angew. Chem. Int. Ed. Engl..

[39] Vosjan MJWD (2010). Conjugation and radiolabeling of monoclonal antibodies with zirconium-89 for PET imaging using the bifunctional chelate p-isothiocyanatobenzyl-desferrioxamine. Nat. Protoc..

[40] Herndler-Brandstetter D (2017). Humanized mouse model supports development, function, and tissue residency of human natural killer cells. Proc. Natl. Acad. Sci. U. S. A..

