# Supplementary material for: Generation of T-cell-redirecting bispecific antibodies with differentiated profiles of cytokine release and biodistribution by CD3 affinity tuning
Source: Sci Rep. 2021 Jul 13;11:14397. doi: 10.1038/s41598-021-93842-0 (PMC8277787; doi:10.1038/s41598-021-93842-0)
Supplement: Supplementary file 1 — Supplementary Information 1. [file 41598_2021_93842_MOESM1_ESM.docx]

**Generation of T-cell redirecting bispecific antibodies with differentiated profiles of cytokine release and biodistribution by CD3 affinity tuning**

Lauric Haber*^1^, Kara Olson^1^, Marcus P. Kelly^1^, Alison Crawford^1^, David J. DiLillo^1^, Richard Tavaré^1^, Erica Ullman^1^, Shu Mao^1^, Lauren Canova^1^, Olga Sineshchekova^1^, Jennifer Finney^1^, Arpita Pawashe^1^, Supriya Patel^1^, Ryan McKay^1^, Sahar Rizvi ^1^, Ermelinda Damko^1^, Danica Chiu^2^, Kristin Vazzana^1^, Priyanka Ram^1^, Katja Mohrs^1^, Amanda D’Orvilliers^1^, Jenny Xiao^1^, Sosina Makonnen^1^, Carlos Hickey^1^, Cody Arnold^1^, Jason Giurleo^1^, Ya Ping Chen^1^, Courtney Thwaites^3^, Drew Dudgeon^1^, Kevin Bray^1^, Ashique Rafique^1^, Tammy Huang^1^, Frank Delfino^1^, Aynur Hermann^1^, Jessica R. Kirshner^1^, Marc W. Retter^1^, Robert Babb^1^, Douglas MacDonald^1^, Gang Chen^1^, William C. Olson^1^, Gavin Thurston^1^, Samuel Davis^1^, John C. Lin^1^, Eric Smith^1^

**Affiliations:**

^1^Regeneron Pharmaceuticals, Inc., Tarrytown, NY 10591, United States

^2^AstraZeneca, Cambridge, UK

^3^Lonza, Slough, UK

*To whom correspondence should be addressed: [Lauric.haber@regeneron.com](mailto:Lauric.haber@regeneron.com)

**Supplementary Figure S1**. **(a)** Representation of bispecific antibody format (left); binding of TSAxCD3 bispecific antibodies to Jurkat cells by flow cytometry (right). **(b)** Purity of a representative panel of bispecific and parental antibodies.

**a**

**b**

| **Protein name** | **Lot#** | **SE-UPLC Main Peak Purity (%) Summary** | **HMW (%)** |
| --- | --- | --- | --- |
| PSMAxCD3^M^ bispecific Ab | L2 | 98.5 | 1.5 |
| PSMAxCD3^W^ bispecific Ab | L2 | 99.4 | 0.6 |
| PSMAxCD3^VW^ bispecific Ab | L3 | 98.2 | 1.9 |
| MUC16xCD3^M^ bispecific Ab | L58 | 98.4 | 1.6 |
| MUC16xCD3^W^ bispecific Ab | L11 | 99.2 | 0.4 |
| MUC16xCD3^VW^ bispecific Ab | L2 | 99.4 | 0.6 |
| CD3 mAb: CD3^M^ | L6 | 98.3 | 1.8 |
| CD3 mAb: CD3^W^ | L3 | 98.2 | 1.2 |
| CD3 mAb: CD3^VW^ | L2 | 98.4 | 1.6 |

**Supplementary Figure S2.** Binding of CD3 bivalent antibodies to Jurkat CD3+ cells and Raji CD3- cells by flow cytometry, and binding of TSAxCD3 bispecific antibodies to control cell lines.

**(a)** Binding of CD3 bivalent antibodies to Jurkat CD3+ cells and Raji CD3- cells by flow cytometry. **(b)** The binding of PSMAxCD3 bispecific antibodies (20 µg/ml) to HEK293 cells engineered to express hPSMA, and control cell lines (TSA-) HEK293, PC-3 and TrampC2 was assessed by flow cytometry (three replicates).

**a**

**b**

**Supplementary Figure S3.** Monovalent and bivalent kinetic binding parameters of CD3^M^, CD3^W^, and CD3^VW^, and binding of PSMAxCD3 bispecific antibodies to C4-2 cells by flow cytometry.

**(a)** The binding kinetics of various CD3 Mabs or TSAxCD3 bispecific antibodies to CD3 was determined by Surface Plasmon Resonance (SPR). hCD3.mFc construct was captured on a Biacore CM5 chip surface immobilized with anti-mIgG2aFc (southern), and serial dilutions of 2 uM stock solutions of antibodies were injected at 37^o^C. Kinetics parameters were evaluated by fitting the real time data using 1:1 binding model with mass transport limitation in Biacore 8k insight software. **(b)** Binding of PSMAxCD3 bispecific antibodies to PSMA+ cells by flow cytometry (data representative of two independent experiments).

**a**

| Antibody | hCD3.Fc Capture (RU) | 2 µM Ab Bind (RU) | Ka (1/Ms) | Kd (1/s) | KD (M) | T1/2 (min) |
| --- | --- | --- | --- | --- | --- | --- |
| Bivalent CD3^M^ | 229.0±7.6 | 34 | 2.85E+05 | 2.06E-03 | 7.25E-09 | 5.6 |
| Bivalent CD3^W^ | 224.1±7.3 | 8.6 | 2.59E+04 | 2.18E-02 | 8.39E-07 | 0.5 |
| Bivalent CD3^VW^ | 226.9±7.5 | 3.8 | 5.40E+04 | 3.40E-02 | 6.30E-07 | 0.3 |
| PSMAxCD3^M^ | 227.9±7.5 | 30.7 | 8.38E+04 | 4.86E-03 | 5.80E-08 | 2.4 |
| PSMAxCD3^W^ | 273.3±26.0 | 2.8 | 3.41E+04 | 2.85E-02 | 8.35E-07 | 0.4 |
| PSMAxCD3^VW^ | 218.9±7.0 | 0.9 | NC | NC | NC | NC |
| Parental PSMA | 226.6±7.5 | -2.0 | NB | NB | NB | NB |
| Isotype control | 261.2±24.1 | -2.7 | NB | NB | NB | NB |

*NC* not calculated, *NB* No binding detected.

**b**

**Supplementary Figure S4**. Human CD3-mediated NFAT signaling in the absence of cells expressing tumor antigen. Data representative of two independent experiments, replicates of three.

JurkatNFATLuc + Control cell line (HEK293)

**Supplementary Figure** **S5.** PD-1 upregulation in a cytotoxicity assay targeting OVCAR-3 cells measured by flow cytometry; cytotoxic potency of bispecific antibodies in the presence of purified T cells; T-cell activation is not observed in the absence of tumor cells expressing the targeted antigen.

**(a)** PD-1 upregulation in a cytotoxicity assay targeting OVCAR-3 cells measured by flow cytometry (data representative of two independent experiments, three replicates). **(b)** The *in vitro* cytotoxic potency of MUC16xCD3 bispecific antibodies against OVCAR-3 was assessed in the presence of PBMC (filled symbols, solid lines) or purified T cells (hollow symbols, dotted lines) from the same human PBMC donor (data representative of two independent experiments, three replicates). **(c)** T-cell activation (CD16-/CD2+ cells) was assessed by flow cytometry in the absence of target cells expressing the tumor antigen. hPBMC and antibodies (or CD3/CD28 activation beads) were co-incubated for 72 hours, and the expression of the activation marker CD25 on T cells was reported as a percentage of CD16-/CD2+/CD25+ T cells out of the T-cell population (data representative of two independent experiments).

**a**

CD8+/PD1+ T cells

**b**

**c**

**Supplementary Figure S6.** Binding to human and cynomolgus BCMA from BCMAxCD3 bispecific antibodies measured by Biacore **(a)**. Binding to human and cynomolgus CD20 from CD20xCD3 bispecific antibodies measured by flow cytometry **(b)**. Data representative of three cynomolgus donors.

CD20xCD3^M^ and CD20xCD3^W^ show equivalent binding to cynomolgus B cells by flow cytometry

**a**

**b**

**Supplementary Figure S7.** CD20+ B-cell depletion in cynomolgus peripheral tissues.

Bispecific T-cell engaging antibodies with different CD3 affinities achieved comparable potency in cynomolgus monkeys in depleting CD20+ B cells in peripheral tissues (spleen, bone marrow, thymus, mesenteric lymph node).

Statistical analysis by ordinary one-way ANOVA with Tukey’s multiple comparisons test. **P<0.007, ****P<0.0001.

**Supplementary Figure S8.** Cytotoxic potency of TSAxCD3 bispecific antibodies targeting OVCAR-3 cells assessed in a time course experiment by flow cytometry.

Briefly, the viability of OVCAR-3 cells was measured in an *invitro* cytotoxicity assay (4:1 E:T ratio) at 72 h and 96 h time points in the presence of a titration of a TSAxCD3 bispecific antibody.

**Supplementary Table S1.** Summary of CD3 binding EC50 [M] by flow cytometry.

|  |  | **Jurkat** | **Human  T cells** | **Cynomolgus  T cells** |
| --- | --- | --- | --- | --- |
| **Monovalent CD3^M^** | TSAx7221G | 1.80E+08 | 3.40E-08 | 3.60E-08 |
| **Monovalent CD3^VW^** | TSAx7221G5 | NC | NC | NC |
|  | TSAx7221G9 | 9.80E-08 | NT | NT |
|  | TSAx7221G14 | 3.10E-08 | NT | NT |
|  | TSAx7221G17 | 1.40E-07 | NT | NT |
| **Monovalent CD3^W^** | TSAx7221G20 | 3.30E-07 | 2.70E-07 | 3.20E-07 |
| **Bivalent CD3^M^** | Bivalent 7221G (moderate) | NT | 3.00E-09 | 7.10E-10 |
| **Bivalent CD3^W^** | Bivalent 7221G20 (weak) | NT | 1.70E-08 | 9.90E-09 |
| **Bivalent CD3^VW^** | Bivalent 7221G5 (very weak) | NT | 2.90E-08 | NC |

*NC* not calculated, *ND* not detected, *NT* not tested.

**Supplementary Table S2a.** Mean pharmacokinetic parameters of total isotype control, total CD20xCD3^M^ and total CD20xCD3^W^ concentrations in serum vs. time following a single intravenous infusion in the male cynomolgus monkey.

| **Parameter** | **Unit** | **Isotype control 0.1 mg/kg** | | | | **CD20xCD3^M^ 0.1 mg/kg** | | | | **CD20xCD3^W^ 0.1 mg/kg** | | | |
| --- | --- | --- | --- | --- | --- | --- | --- | --- | --- | --- | --- | --- | --- |
|  |  | **N** | **Mean** | **SD** | **CV%** | **N** | **Mean** | **SD** | **CV%** | **N** | **Mean** | **SD** | **CV%** |
| C_max_ | µg/mL | 4 | 2.57 | 0.114 | 4.45 | 8 | 2.6 | 0.425 | 16.3 | 8 | 2.55 | 0.23 | 9.03 |
| C_max_/Dose | (µg/mL)/(mg/kg) | 4 | 25.7 | 1.14 | 4.45 | 8 | 26 | 4.25 | 16.3 | 8 | 25.5 | 2.3 | 9.03 |
| t_max_ | h | 4 | 0.688 | 0.208 | 30.3 | 8 | 0.600 | 0.0267 | 4.45 | 8 | 0.598 | 0.0226 | 3.78 |
| AUC_last_ | Day*µg/mL | 2 | 20.1 | NC | NC | 4 | 4.43 | 1.40 | 31.6 | 4 | 10.9 | 2.09 | 19.1 |
| AUC^inf^ | Day*µg/mL | 2 | 24.3 | NC | NC | 4 | 4.65 | 1.35 | 29.0 | 4 | 12.2 | 2.05 | 16.8 |
| AUC_last_/Dose | (Day*µg/mL)/(mg/mL) | 2 | 201 | NC | NC | 4 | 44.3 | 14.0 | 31.6 | 4 | 109 | 20.9 | 19.1 |
| AUC_inf_/Dose | Day*kg*µg/mL/mg | 2 | 243 | NC | NC | 4 | 46.5 | 13.5 | 29.0 | 4 | 122 | 20.5 | 16.8 |
| AUC_inf% Extrapolated_ | % | 2 | 16.9 | NC | NC | 4 | 5.37 | 2.96 | 55.2 | 4 | 10.6 | 10.3 | 96.6 |
| t_1/2_ | Day | 2 | 13.8 | NC | NC | 4 | 2.54 | 0.497 | 19.6 | 4 | 6.18 | 3.17 | 51.3 |
| CL | mL/day/kg | 2 | 4.14 | NC | NC | 4 | 23.1 | 7.55 | 32.6 | 4 | 8.38 | 1.54 | 18.4 |
| V_ss_ | mL/kg | 2 | 76.6 | NC | NC | 4 | 71.9 | 9.48 | 13.2 | 4 | 76.2 | 16.9 | 22.2 |

*AUC_inf_* area under the concentration–time curve from time zero extrapolated to infinity, *AUC_inf% Extrapolated_* percentage of the area under the concentration–time curve extrapolated in the terminal phase to AUC_inf_, *AUC_last_* area under the concentration–time curve computed from the time of dosing to the time of the last measurable concentration, *CL* total body clearance, *C_max_* peak concentration, *CV* coefficient of variation, *N* number of animals, *PK* pharmacokinetics, *SD* standard deviation, *t_1/2_* half-life, *t_max_* time to C_max_, *V_ss_* volume of distribution at steady state.

**Supplementary Table S2b.** Mean pharmacokinetic parameters of total BCMAxCD3^M^ and total BCMAxCD3^W^ in serum following a single intravenous injection of BCMAxCD3^M^ and BCMAxCD3^W^ in the male cynomolgus monkey.

| **BCMAxCD3^M^ (N=3)** | | | | | | | | | | |
| --- | --- | --- | --- | --- | --- | --- | --- | --- | --- | --- |
| **Parameter** | **Unit** | **BCMAxCD3^M^ 0.1 mg/kg** | | | **BCMAxCD3^M^ 1 mg/kg** | | | **BCMAxCD3^M^ 5 mg/kg** | | |
|  |  | **Mean** | **SD** | **CV%** | **Mean** | **SD** | **CV%** | **Mean** | **SD** | **CV%** |
| C_max_ | µg/mL | 2.66 | 0.311 | 11.7 | 29.9 | 3.84 | 12.9 | 142 | 4.36 | 3.07 |
| C_max_/dose | (µg/mL)/(mg/kg) | 26.6 | 3.11 | 11.7 | 29.9 | 3.84 | 12.9 | 28.4 | 0.872 | 3.07 |
| t_max_ | H | 0.0833 | 1.01E-10 | 1.21E-07 | 0.0833 | 1.01E-10 | 1.21E-07 | 0.0833 | 1.01E-10 | 1.21E-07 |
| AUC_last_ | Day*µg/mL | 12.0 | 3.24 | 27.0 | 118 | 41.5 | 35.2 | 582 | 51.4 | 8.83 |
| AUC_inf_ | Day*µg/mL | 12.2 | 3.35 | 27.5 | 120 | 42.7 | 35.6 | 601 | 60.5 | 10.1 |
| AUC_last_/dose | Day*(µg/mL)/(mg/mL) | 120 | 32.4 | 27.0 | 118 | 41.5 | 35.2 | 116 | 10.3 | 8.83 |
| AUC_inf_/dose | Day*(µg/mL)/(mg/mL) | 122 | 33.5 | 27.5 | 120 | 42.7 | 35.6 | 120 | 12.1 | 10.1 |
| AUC_inf% extrapolated_ | % | 1.50 | 1.08 | 71.6 | 1.62 | 1.49 | 92.1 | 3.07 | 3.41 | 111 |
| t_1/2_ | Day | 5.17 | 2.99 | 57.8 | 4.38 | 2.61 | 59.5 | 4.45 | 2.57 | 57.7 |
| CL | mL/day/kg | 8.58 | 2.04 | 23.8 | 9.08 | 3.27 | 36.0 | 8.38 | 0.879 | 10.5 |
| V_ss_ | mL/kg | 62.7 | 7.95 | 12.7 | 57.4 | 3.28 | 5.71 | 58.5 | 10.5 | 18.0 |

| **BCMAxCD3^W^ (N=3)** | | | | | | | | | | |
| --- | --- | --- | --- | --- | --- | --- | --- | --- | --- | --- |
| **Parameter** | **Unit** | **BCMAxCD3^W^ 0.1 mg/kg** | | | **BCMAxCD3^W^ 1 mg/kg** | | | **BCMAxCD3^W^ 5 mg/kg** | | |
|  |  | **Mean** | **SD** | **CV%** | **Mean** | **SD** | **CV%** | **Mean** | **SD** | **CV%** |
| C_max_ | µg/mL | 2.49 | 0.667 | 26.8 | 27.0 | 4.70 | 17.4 | 126 | 22.3 | 17.8 |
| C_max_/Dose | (µg/mL)/(mg/kg) | 24.9 | 6.67 | 26.8 | 27.0 | 4.70 | 17.4 | 25.1 | 4.47 | 17.8 |
| t_max_ | h | 1.42 | 2.31 | 163 | 0.0833 | 1.01E-10 | 1.21E-07 | 0.0833 | 1.01E-10 | 1.21E-07 |
| AUC_last_ | Day*µg/mL | 28.1 | 4.17 | 14.8 | 294 | 55.5 | 18.9 | 953 | 311 | 32.7 |
| AUC^inf^ | Day*µg/mL | 35.1 | 6.68 | 19.0 | 357 | 81.7 | 22.9 | 1410 | 815 | 57.7 |
| AUC_last_/Dose | Day*(µg/mL)/(mg/mL) | 281 | 41.7 | 14.8 | 294 | 55.5 | 18.9 | 191 | 62.3 | 32.7 |
| AUC_inf_/Dose | Day*(µg/mL)/(mg/mL) | 351 | 66.8 | 19.0 | 357 | 81.7 | 22.9 | 283 | 163 | 57.7 |
| AUC_inf% Extrapolated_ | % | 19.4 | 4.53 | 23.4 | 17.0 | 4.45 | 26.2 | 25.0 | 22.4 | 89.6 |
| t_1/2_ | Day | 18.2 | 2.45 | 13.5 | 17.0 | 1.75 | 10.3 | 13.4 | 10.7 | 79.4 |
| CL | mL/day/kg | 2.92 | 0.562 | 19.3 | 2.91 | 0.680 | 23.4 | 4.37 | 2.27 | 51.8 |
| V_ss_ | mL/kg | 72.9 | 6.60 | 9.05 | 67.3 | 10.7 | 15.9 | 69.8 | 25.3 | 36.2 |

*AUC_inf_* area under the concentration–time curve from time zero extrapolated to infinity, *AUC_inf% Extrapolated_* percentage of the area under the concentration–time curve extrapolated in the terminal phase to AUC_inf_, *AUC_last_* area under the concentration–time curve computed from the time of dosing to the time of the last measurable concentration, *CL* total body clearance, *C_max_* peak concentration, *CV* coefficient of variation, *N* number of animals, *PK* pharmacokinetics, *SD* standard deviation, *t_1/2_* half-life, *t_max_* time to C_max_, *V_ss_* volume of distribution at steady state.

**Supplementary Table S3.** Summary of safety assessment for the CD20xCD3 and BCMAxCD3 cynomolgus studies.

| **Study** | **Observations** |
| --- | --- |
| CD20xCD3 | 3 animals exhibited vomitus and 1 animal had liquid feces on Day 1 following dosing of CD20xCD3^M^. An increase in C-reactive protein determined by clinical chemistry analysis correlated with the cytokine increases that were noted for both CD20xCD3^M^ and CD20xCD3^W^ groups, but the changes were less than 5-fold compared to the levels observed in the isotype control group. No changes in body temperature were noted for either the CD20xCD3^M^ or CD20xCD3^W^ groups following dosing on Day 1 |
| BCMAxCD3 | Animals administered ≥ 1 mg/kg BCMAxCD3^M^ and 1 animal administered 5 mg/kg BCMAxCD3^W^ exhibited vomitus on Day 1 following dosing, which also correlated with a mild increase (approximately 10-fold compared to pre-dose levels) in  C-reactive protein in these same groups. No changes in body temperature were noted for either the BCMAxCD3^M^ or BCMAxCD3^W^ groups following dosing |

*m* moderate; *w* weak.

**Supplementary Table S4.** Amino acid sequence of 7221G variants generated by reversing amino acids to the corresponding germline residues.

**Supplementary methods:**

**Nuclear factor of activated T cells-luciferase (NFAT-luc) cell-based reporter bioassay**

A luciferase reporter bioassay was developed in Jurkat T cells that were stably transfected with an NFAT response element-luciferase reporter plasmid (Jurkat/NFAT-luc). Activation of NFAT, via CD3 engagement with serial dilutions of TSAxCD3 or control CD3 antibodies was evaluated in the presence of MUC16+ OVCAR-3 cells, PSMA+C4-2 cells, CD20+ RAJI cells (where CD80 and CD86 expression was eliminated using CRISP/Cas9), and BCMA-expressing H929 cells.

Serial dilutions of TSAxCD3 or CD3-binding control antibodies were added to Jurkat/NFAT-luc cells (50,000 cells/well) in the presence of TSA-expressing cells (50,000 cells/well). The antibody dilutions and bioassay were performed in Jurkat complete media (RPMI medium 1640 supplemented with 10% FBS and 1X penicillin-streptomycin L-glutamine). Wells containing no antibody were used as a control. Plates were incubated at 37°C, 5% CO_2_ for 5 hours and then brought to room temperature for 15 minutes. One-Glo luciferase substrate (100 μL) was added to each well, and the plates were incubated for 3 minutes. The luminescence signal was measured using the ENVISION plate reader, and measured values were analyzed by a four‑parameter logistic equation over a multi-point response curve using GraphPad Prism version 7.0e for Mac OS X, GraphPad Software, SanDiego, California USA, www.graphpad.com.

***In vitro* flow cytometry cytotoxicity assays using human effector cells**

For the hematologic tumor cell lines study, human PBMCs were thawed and plated in complete media at 1x10^6^ cells/mL and incubated overnight at 37°C in order to enrich for lymphocytes by depleting adherent cells. The following day, MOLP-8 cells were labeled with 1 μM of Violet Cell Tracker fluorescent tracking dye. Labeled MOLP-8 cells (1x10^4^ cells/well) were plated in round-bottom 96-well plates at an effector:target ratio of 4:1 with non-adherent PBMCs and serial dilutions of BCMAxCD3 bsAb or CD3-binding control bsAb for 48 hours at 37°C in complete media. At the end of the culture, surviving target cells and T-cell activation were analyzed on a BD FACSCelesta flow cytometer. For analysis by flow cytometry, cells were washed with cold PBS and stained with a LIVE/DEAD Fixable Aqua Dead Cell Stain to identify viable cells. For assessment of MOLP-8 killing, cells were gated on live Violet-labeled populations. The percent live population was recorded and used for the calculation of survival. Percent viability was normalized to control condition (target cells in the presence of PBMCs only).

T-cell activation was assessed by incubating cells with an antibody phenotyping cocktail containing PE-conjugated anti-CD8 mAb, APC-H7-conjugated anti-CD4 mAb, FITC-conjugated anti-CD2 mAb, and APC-conjugated anti-CD25 mAb in Miltenyi AutoMacs Wash for 45 minutes at 4°C. Cells were then washed twice and re-suspended in cold PBS containing 1% filtered FBS and analyzed by flow cytometry on a BD FACSCelesta flow cytometer. Activation is reported as the percentage of CD2^+^/CD8^+^ T cells expressing CD25. Percent T-cell activation was normalized to control condition (target cells in the presence of PBMCs only).

**Parental blocking cytotoxicity assay**

A flow cytometry-based approach was used to determine the ability of parental PSMA or MUC16 antibodies to block the ability of the PSMAxCD3 or MUC16xCD3 bsAbs to mediate tumor cell lysis. PSMA+ C4-2 or MUC16+ OVCAR-3 tumor cell lines were labeled with 1 μM Violet Cell Tracker. After labeling, the cells were counted, and 7.5 × 10^4^ cells were plated in 96-well flat bottom plates for overnight culture at 37°C. Separately, isolated human PBMCs were plated in complete RPMI media at 1 × 10^6^ mL and incubated overnight at 37°C in order to enrich T cells. The following day, T-cell-enriched naïve human PBMCs were stained with 1 μM CFDA-SE (Life Sciences) and 3 × 10^4^ cells were added to the plated target cells (with a 4:1 ratio of effector to tumor cells). Four different fixed concentrations of parental PSMA or MUC16 antibodies (1 ug/mL, 0.1 ug/mL, and 0.01 ug/mL, and 0 ug/mL=no block) were added, followed by a fixed concentration (50 pg/mL) of the different PSMAxCD3 or MUC16xCD3 bsAbs. The plates were incubated at 37°C for 72 hours. After incubation, effector and target cells were removed from the plate and analyzed by flow cytometry. For studying the viability of the tumor cells, cells were stained with a cocktail of labeled antibodies and LIVE/DEAD Near-IR dye following the manufacturer’s protocols (Invitrogen). Statistical analysis was performed using GraphPad Prism version 7.0e for Mac OS X, GraphPad Software, SanDiego, California USA, [www.graphpad.com](http://www.graphpad.com). Statistical significance for C4-2 or OVCAR-3 percent viability was determined by two-way analysis of variance (ANOVA) with Tukey’s multiple comparisons post-test.

**Cynomolgus pharmacokinetic studies**

***Animals***

- BCMAxCD3 study: Covance, males, 2.5–3.5 years of age, 2.4–3.8 kgs; Certified Primate Diet #5048 (PMI Nutrition International Certified LabDiet).
- CD20xCD3 study: Charles River Labs, males (obtained from Covance), 2–4 years of age, 2.8–3.5 kgs; Certified Primate Diet# 5048 (PMI Nutrition International Certified LabDiet).

Male cynomolgus monkeys were obtained from Covance Laboratories. Monkeys were 2–4 years of age (2.4–3.8 kg). Animals were housed in stainless steel cages equipped with a stainless-steel mesh floor. Environmental controls were set to maintain the following animal room conditions: temperature range of 18–29°C, relative humidity range of 30–70%, 10 or more air changes/hour and a 12-hour light/12-hour dark cycle. The light/dark cycle was interrupted for study-related activities. Animals were given various cage-enrichment devices and fruit, vegetable, or dietary enrichment. Animals were commingled in accordance with Covance standard operating procedures. Animals were offered Certified Primate Diet #5048 (HPMI Nutrition International) one to two times daily unless fasted for study procedures and water was supplied to animals *ad libitum.* Veterinary care was available throughout the course of the study and animals were examined by the veterinary staff as warranted by clinical signs or other changes. All procedures in the protocol were in compliance with applicable animal welfare acts, were approved by the local IACUC, and were carried out in accordance with the approved guidelines.

***Cynomolgus monkey study design***

BCMAxCD3: 21 male cynomolgus monkeys were assigned to one of seven groups (three animals/group). Animals received a single IV bolus dose of saline, or 0.1, 1, or 5 mg/kg BCMAxCD3^M^ or BCMAxCD3^W^. Blood samples were collected from all animals from pre-dose through 648 hours (27 days, 5 mg/kg groups) or 1,008 hours (42 days, 0.1 and 1 mg/kg groups) post-dose to determine the concentrations of total BCMAxCD3^M^ and total BCMAxCD3^W^ in serum.

CD20xCD3: 20 male cynomolgus monkeys were assigned to one of three groups (four animals/group for the isotype control, and eight animals/group for CD20xCD3^M^ and CD20xCD3^W^). Animals received a single IV bolus dose of 0.1 mg/kg of an isotype control bsAb, CD20xCD3^M^, or CD20xCD3^W^. Blood samples were collected from all animals from pre-dose through 840 hours (35 days) post-dose to determine the concentrations of total CD20xCD3^M^ and total CD20xCD3^W^ in serum.

Cytokines were measured by MSD.

Circulating B-cell depletion and bone marrow plasma-cell depletion in the CD20xCD3 and BCMAxCD3 studies, respectively, was assessed using flow cytometry. Briefly, for the CD20xCD3 study, whole blood was collected in K_2_EDTA, lysed, stained for CD45, CD19, CD20, CD3, and CD16 and the percentage of B cells (CD45+/CD20+/CD3-) of the lymphocyte gate was determined using a FACS Canto II (BD). Absolute B-cells counts were calculated using the lymphocyte counts generated by the concurrent hematology assessments. For the BCMAxCD3 study, bone marrow aspirate was collected in sodium heparin tubes, red blood cells were lysed, and remaining lymphocytes were stained for CD45, CD3, CD27, CD38, CD20, CD19, and CD138 (BD Biosciences). Relative percentage of bone marrow stem plasma cells (CD3-CD45+CD27+CD38+CD138+) were determined using a fortessa flow cytometer (BD).

***Total TSAxCD3^M^ and TSAxCD3^W^ in Monkey Serum***

Concentrations of total BCMAxCD3^M^ and BMCAxCD3^W^ or CD20xCD3^M^ and CD20xCD3^W^ in cynomolgus monkey serum were measured using a non-validated, enzyme-linked immunosorbent assay (ELISA). The lower limit of quantification (LLOQ) is 0.0078 μg/mL in neat monkey serum.

This ELISA method employs microtiter plates coated with a mouse anti-human Ig, kappa light chain specific monoclonal antibody as the capture reagent. TSAxCD3^M^, TSAxCD3^W^, or an isotype control captured on the plate is detected using a biotinylated mouse anti-human IgG Fc* monoclonal antibody (specific for a 2 amino acid substitution [Fc*] engineered into the Fc domain of one of the heavy chains of bsAbs[37], followed by Neutravidin conjugated with horseradish peroxidase. A luminol-based substrate specific for peroxidase is then added to generate a signal whose intensity is proportional to the concentration of total TSAxCD3^M^, TSAxCD3^W^, or isotype control.

Statistical analysis by ordinary one-way ANOVA with Tukey’s multiple comparisons test using GraphPad Prism version 7.0e for Mac OS X, GraphPad Software, SanDiego, California USA, [www.graphpad.com](http://www.graphpad.com).

***Pharmacokinetic analysis***

Concentrations of total TSAxCD3^M^ and total TSAxCD3^W^ were analyzed by noncompartmental analysis (NCA) using Phoenix, WinNonlin (Version 6.4, Certara, L.P, www.certara.com) using an IV bolus (BCMA) or IV infusion (CD20) models. The target doses of TSAxCD3^M^ or TSAxCD3^W^, as described in the study protocol, were used for NCA. The actual administered doses were within 10% of the target dose.

**Binding kinetics by surface plasmon resonance**

***BCMA binding kinetics by*** ***surface plasmon resonance***

The binding kinetics of BCMAxCD3 bsAbs to human and monkey monomeric BCMA proteins (hBCMA.mmH and mfBCMA.mmH, respectively) were measured on a Biacore T200 instrument. Briefly, mouse anti-human Fc monoclonal antibody (Jackson ImmunoResearch or REGN2567) was immobilized on the surface of a CM5 sensor chip using standard EDC/NHS amine-coupling. At 25°C or 37°C using HBS-EP running buffer (10 mM HEPES, 150 mM NaCl, 3 mM EDTA, 0.05% polysorbate 20, pH 7.4), approximately 50–100 RU of BCMAxCD3 bsAbs were captured onto this surface. Recombinant human and monkey BCMA proteins (2-fold serial dilutions at concentrations ranging from 0.2 nM to 25 nM or 1.56 nM to 200 nM, hBCMA.mmH or mfBCMA.mmH, respectively) were individually injected at 50 μL/min for 5 minutes followed by a 10-minute dissociation phase. All concentrations were tested in duplicate.

***CD3 binding kinetics by surface plasmon resonance***

Binding kinetics of heterodimeric human CD3d/CD3e protein to anti-CD3-containing antibodies were measured on a Biacore 8k instrument. Briefly, polyclonal goat anti-mouse IgG2a Fc Ab (Southern Biotech) was immobilized on the surface of a CM5 sensor chip using standard EDC/NHS amine-coupling. At 25°C or 37°C using HBS-EP running, approximately 250 RU of human CD3 protein was captured onto this surface. Anti-CD3 antibodies (4-fold serial dilutions ranging from 1.95 nM to 2,000 nM) were individually injected at 50 μL/min for 4 minutes followed by a 5-minute dissociation phase. All concentrations were tested in duplicate.

***Surface plasmon resonance data analysis***

Specific SPR sensorgrams were obtained by a double referencing procedure by first subtracting the signal of each injection over a reference surface from the signal over the experimental surface, thereby removing contributions from refractive index changes. In addition, running buffer injections were performed to allow subtraction of the signal changes resulting from the dissociation of captured protein from the sensor surface. The kinetic parameters were obtained by globally fitting these specific sensorgrams to a 1:1 binding model with mass transport limitation using Biacore T200 Evaluation software v2.0, www.biacore.com, or Scrubber v2.0c, Biologic software, Canberra, Australia, www.biologic.com.au. The equilibrium dissociation constant (K_D_) was calculated from the ratio of the dissociation rate constant to the association rate constant (K_D_ = *k*_d_/*k*_a_). The dissociative half-life was calculated by dividing 0.693 (natural logarithm of 2) by the experimentally determined *k*_d_.
